# How experienced robotic nurses adapt to the Hugo™ RAS system

**DOI:** 10.1007/s11701-024-01878-x

**Published:** 2024-03-11

**Authors:** Rikke Groth Olsen, Flemming Bjerrum, Lars Konge, Julia Abildgaard Dagnæs-Hansen, Louise Møller, Nana Levann, Didde Barfred, Andreas Røder

**Affiliations:** 1 Department of Urology, Copenhagen Prostate Cancer Center, Copenhagen University Hospital-Rigshospitalet, Ole Maaløes Vej 24, 2200 Copenhagen, Denmark; 2 Copenhagen Academy for Medical Education and Simulation (CAMES), Copenhagen, Denmark; 3 Faculty of Health and Medical Sciences, University of Copenhagen, Copenhagen, Denmark; 4 Gastrounit, Surgical Section, Copenhagen University Hospital–Amager and Hvidovre, Hvidovre, Denmark; 5 Department of Anaesthesiology, Centre for Cancer and Organ Disease, Copenhagen University Hospital–Rigshospitalet, Copenhagen, Denmark

**Keywords:** Robotic surgery, Nurses, Team, Learning curve, Hugo^™^ RAS

## Abstract

No studies have reported on the impact at team level of the Medtronic Hugo^™^ RAS system. We described the work patterns and learning curves of an experienced robotic nurse team adapting to the new robotic system. We prospectively recorded the robotic nurse team’s preoperative, perioperative, and postoperative tasks on the first 30 robotic procedures performed. The data were descriptively analyzed, and Gantt Charts were created for a timeline overview of the work patterns. We compared the operative times between the Medtronic Hugo^™^ RAS and the Davinci^®^ system. The preoperative phase seemed to improve with a median time of 94 min (IQR 81–107). After 20 surgeries, the work pattern became more consistent where the scrub and circulating nurses worked simultaneously. There was no noticeable improvement for the perioperative and postoperative phases with a stable median time of 170 min (IQR 135–189) and 26 min (IQR 22–31). We found that the work pattern seemed to stabilize after 20 surgeries but with a continued decrease in preoperative time without a learning curve plateau. The robotic nurse team suffered from few breaks and long working hours because only a few nurses at our facility were trained in the Hugo^™^ system.

**Supplementary Information:**

The online version contains supplementary material available at 10.1007/s11701-024-01878-x.

## Introduction

At the beginning of the twenty first century, robotic surgery made its entrance into the operating room (OR) and changed the responsibilities and competencies required of OR nurses. Robotic nurses have multiple tasks, preoperative, perioperative, and postoperative. They must prepare and check the system, position and secure the safety of the patient, unpack the correct equipment, help the surgeon, pay attention to the rules of sterile and unsterile parts of the robot, and read and interpret errors from the robotic system. Surgeons today are dependent on robotic nurses having the necessary technical knowledge to solve the technical challenges that may arise during robotic surgery [1–3].

The demands for robotic surgeries have increased in recent years, as multiple different robotic systems are now available [4]. The first robotic system, the DaVinci^®^ Surgical System, was implemented in the early 2000. It has a closed surgeon console with binoculars allowing for three-dimensional vision, two joysticks to control the movement of the robotic surgical arms, and foot pedals to control the camera’s movement and activate electrocautery. A movable arm cart with four robotic arms is placed next to the operating bed [5]. In early 2021, the Medtronic Hugo^™^ RAS was introduced. It has an “open console” with a 3D screen, the surgeons wear 3D glasses, have pistol-like joysticks for movement of the robotic surgical arms, and foot pedals to control the camera’s movement and activate electrocautery. The four robotic arms are on individual, movable arm carts which are placed next to the operating bed [6].

Since the introduction of the new robotic system, Medtronic Hugo^™^ RAS, more than 25 studies have been published examining surgical data and short-term patient outcomes; however, no studies have reported on the impact or performance at the team level and how introducing a new robotic system affects the work of the OR team [7]. The safety and efficacy of robot-assisted surgery depend on the surgical team’s experience in the robotic system used. At the beginning of the team’s learning curve, we expect a prolonged operative time and a higher risk of errors. This transition between surgical systems has previously been associated with an increase in complications [2, 8–11].

We aimed to explore the workflow and learning curves of an experienced robotic OR nurse team adapting to the new robotic system, Medtronic Hugo^™^ RAS.

## Methods

We performed a prospective, descriptive study. We have adhered to relevant EQUATOR guidelines and reported by the STROBE reporting guidelines [12] (Supplementary File S1).

In April 2022, Copenhagen University Hospital—Rigshospitalet, Denmark, started using the Medtronic Hugo^™^ RAS for robot-assisted radical prostatectomies (RARP). One experienced RARP surgeon (> 1000 RARPs) transferred from the Davinci^®^ Surgical System to the Hugo^™^. The robotic nurse team was assigned ad hoc from eight experienced robotic nurses and consisted of a circulating nurse, a scrub nurse, and sometimes a supervised circulating nurse in training for each operation. The tasks of the nurses were the same on the Hugo^™^ as for the previous RARP on the DaVinci^®^.

The robotic nurse team received a training program for the Hugo^™^ at Orsi Academy, Belgium, and Copenhagen Academy for Medical Education and Simulation (CAMES), Copenhagen before the first surgery. The robotic team had dry-run team training learning the modalities of the robotic system including set-up, optimal patient positioning, docking, and undocking of the system. They received training to manage technical errors and emergency procedures, including safe and quick undocking of the system.

An engineer and two start-up specialists with OR nursing backgrounds from Medtronic (Minneapolis, Minnesota, USA) were present in the OR for all surgeries to help guide and navigate the robotic system in case of technical issues.

Data from all surgeries with the new system were prospectively registered by a dedicated on-site observer from an observation team of three different observers who all received the same training. All data were observational data with no interventions or extra tasks assigned to the nurses during this study. Data were collected from when the first member of the robotic nurse team entered the OR until the patient had finished surgery and left the OR. The observer noted when the patient and the robotic nurse team were present at the OR and when the robotic nurse team members left the room or were released for a break. Further, data consisted of preoperative, perioperative, and postoperative tasks with the specifications of the tasks explained in Table 1. All data were registered with a start time and end time with a total time calculated from these. The nurses occasionally attended a weekly scheduled educational session during the preoperative phase.

**Table 1:** Descriptions of the tasks of the robotic nurse team

**Preoperative**	**Nurses released for a break** Whenever either the scrub nurse, circulating nurse, or supervised circulating nurse were released for a break by another colleague
	**Nurses leave the OR** When at least one nurse left the OR room to pick up extra equipment, welcomed the patient to the OR, prepared and put on sterile clothing, left for their weekly educational session
	**Starting the robotic system** Connection of the power cords between the arms and the tower, starting the system
	**Unpacking of equipment** Locating and unpacking all sterile and unsterile equipment to be used for the surgery
	**Draping of the robotic arms**^a^ Draping each arm individually with a plastic bag and an interface module for the instruments
	**Positioning the patient**^b^ Safety of the patient with the positioning of the arms and legs, placement of a protective screen over the face of the patient, removal of the clothes, and covering the patient with a heating blanket
	**Preparation of the surgical field**^a^ Sterilization of the surgical field with iodine, application of the sterile garment to the operating bed, and the connection of all power cords between the instruments and the tower
	**Technical errors** Recalibration of one of the robotic arms, restart of the system, change of instrument due to malfunction
**Perioperative**	**Port placement**^a^ Handing instruments to the bedside assistant and surgeon, from the first incision until docking of the first arm
	**Docking of the system** Assisting the bedside assistant and surgeon with positioning the arm cart and instrument insertion
	**Console time**^a^ From docking of the system to undocking of the system
	**Instrument change**^a^ Change of the instrument either due to a technical error or due to the normal progress of the procedure in collaboration with the bedside assistant
	**Rinse of camera lens**^a^ Clean and apply anti-fog to the camera lens in collaboration with the bedside assistant
	**Technical errors** Recalibration of one of the robotic arms, restart of the system, change of instrument due to malfunction
	**Undocking of the system** Undocking of the instruments and arm carts, and removal of them from the operating bed
	**Skin closure**^a^ Assisting the bedside assistant and surgeon with instruments
**Postoperative**	**Equipment cleanup** Removal of the used equipment, turning off the system, disconnection of the power cords
	**Preparing the patient for awakening** Clean up and bandage the surgical wounds, remove the sterile garment of the operating bed, dress the patient

^a^*Performed only by the scrub nurse*

^b^*Performed only by the circulating nurse or supervised circulating nurse*

The Hugo^™^ system was moved from one OR to another after 15 surgeries as the robotic system was first placed at a temporary location while the permanent OR was under renovation. Both ORs were in different buildings away from the regular OR facility of the robotic nurse teams.

For comparison, the same observational data was collected from the experienced robotic team on 30 RARP performed on the DaVinci^®^ Surgical System. The experienced robotic nurse team on the DaVinci^®^ consisted of the same nurses as on the robotic nurse team for the Hugo^™^.

### Ethics

All participants gave oral consent after receiving information about the study. The study was reviewed and approved by The Danish Data Protection Agency (P-2022-341). No further approval was needed from the ethical committee as this was a qualitative assurance study.

### Data analysis

We used descriptive statistics to summarize the basic demographics of the surgeries and the experience of the robotic nurse team. Categorical variables are described by frequencies and proportions while continuous variables are described by median and interquartile range. Total preoperative, perioperative, and postoperative surgical times were calculated and illustrated in a graph for learning curve analysis. The total preoperative surgical times were calculated by extracting the weekly educational session. Timelines were created for a timeline overview of the work patterns for each surgery.

SPSS (Version 28.0.; IBM SPSS Statistics for Windows, Armonk, NY) and Microsoft^®^ Excel^®^ (Version 2202; Microsoft Corporation, Redmond, Washington) were used for the statistical analysis.

## Results

**Fig. 1:**
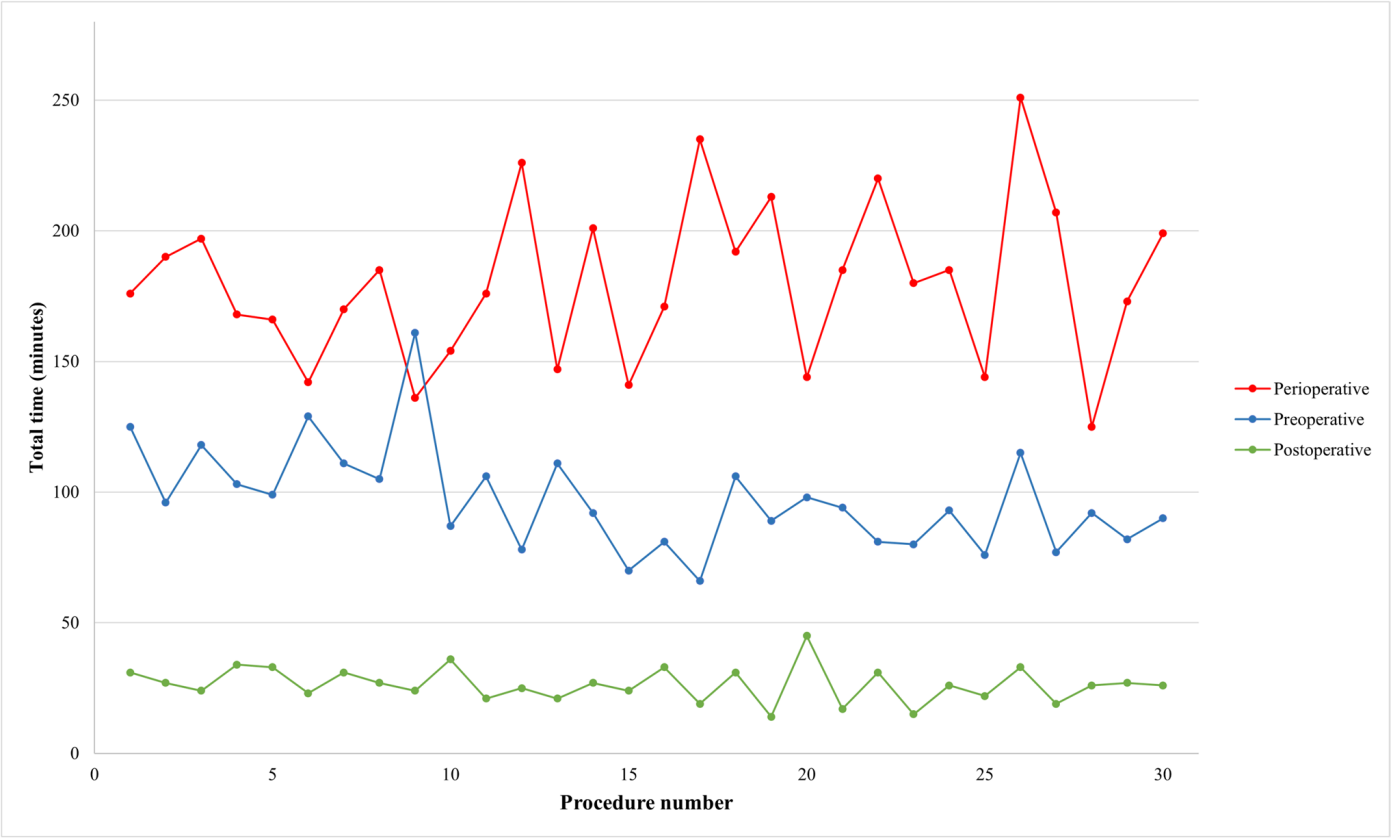
The learning curves for the three surgical phases: preoperative, perioperative, and postoperative times

Thirty RARPs were performed in the study period. Table 2 shows the demographics of the robotic nurse team and the preoperative, perioperative, and postoperative times. Figure 1 and Table 3 show there were no noticeable learning curves for the perioperative and the postoperative phases with stable median surgery time of 178 min (IQR 152–200), and 26 min (IQR 22–31). The preoperative phase improved over time with a median surgery time of 94 min (IQR 81–107) without a certain learning curve plateau.

**Table 2:** Demographics of the robotic nurse team and the preoperative, perioperative, and postoperative tasks

Number of nurses, *n*	8
Years of experience, median years (IQR*)*	4.5 (3–15)
Sex, *n*	
Female	7
Male	1
Number of different team compositions, *n*	15
Total time nurses in the OR room, median time in minutes (IQR)	
Scrub nurse	321 (290–357)
Circulating nurse 1	343 (312–381)
Supervised circulating nurse^a^	372 (304–392)
Number of surgeries the nurses were released for a break, *n *(%)	13 (43%)
Times nurses left the OR room, median* n *(IQR)	4 (3–5)
Surgeries with technical errors, *n *(%)	12 (40%)

^a^*Only present for five surgeries*

**Table 3:** Comparison of surgical procedure times between the Medtronic Hugo^™^ RAS and DaVinci^®^ Surgical system

	Preoperative	Perioperative	Postoperative	Total procedure time
Hugo^™^, median min (IQR)	94 (81–107)	178 (152–200)	26 (22–31)	305 (284–320)
DaVinci^®^, median min (IQR)	68 (47–80)	145 (120–195)	24 (20–29)	243 (207–286)

A significant difference in preoperative time was found between the Hugo^™^ and the DaVinci^®^ (median 94 min vs. 68 min, *p* < 0.001) (Table 3). The preoperative times for Hugo^™^ were slower in starting the robotic system (median + 9 min), unpacking of equipment (median + 10 min), and draping of the robotic arms (median + 8 min) compared to the DaVinci^®^ but were faster for positioning of the patient (median -9 min) and similar in preparation of the surgical field (median ± 0 min). Peri, post, and total procedure time were similar between the systems.

Eight nurses participated in the study with fifteen different team compositions. One team was present for ten surgeries and the rest of the teams were present for between one to three surgeries. All members of the robotic nurse team were present for more than a median of 5 h at the OR. Before the move of the Hugo^™^ system, the circulating nurse was released for a break in 60% of the surgeries and after the move for 27%. The scrub nurse was present for the entire surgery without breaks.

**Fig. 2:**
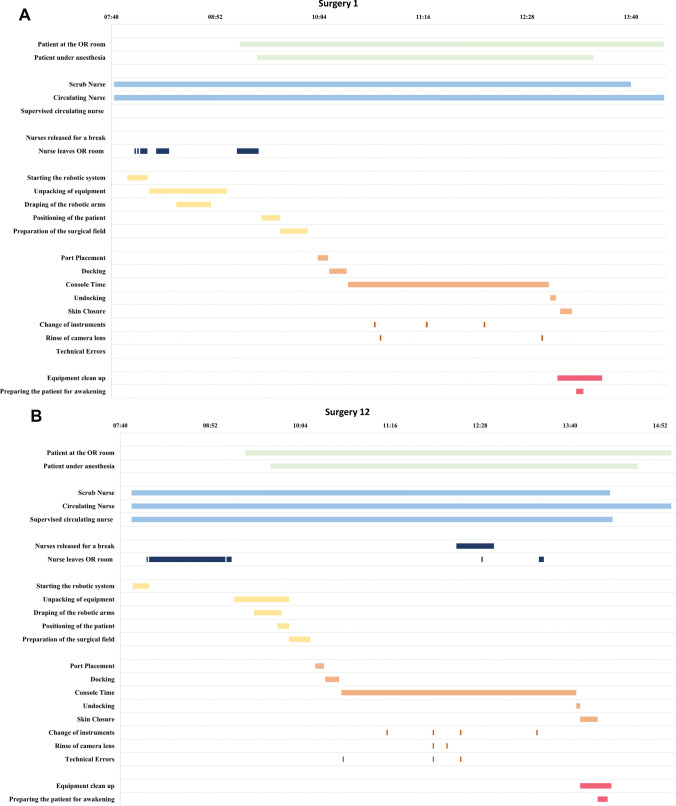

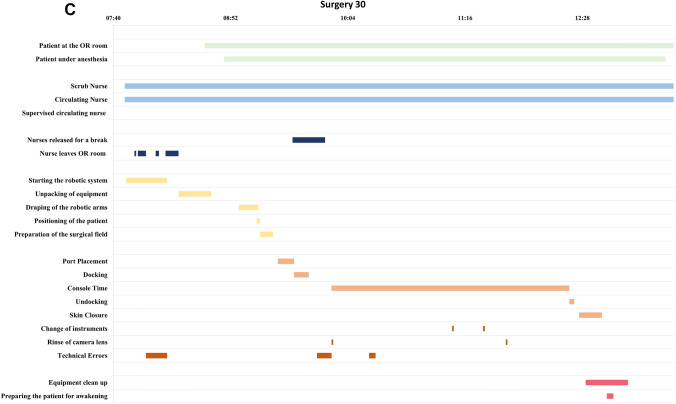
Timelines of the work pattern of the robotic nurse team. **A** The very first surgery with very little overlap of the tasks in the preoperative phase. **B** The pattern of a day with a weekly education session. The system is started before attending the session. Here, the team began to overlap more of the preoperative tasks. **C** A technical error in the starting of the system resulting in a pause before beginning the rest of the preoperative tasks

The timelines show the work patterns of the robotic nurse team (see selected examples in Fig. 2 and all timelines in Supplementary Fig. 1).

In the preoperative phase, the robotic system was started as soon as the robotic nurse team arrived at the OR before any other task was performed. For the first five surgeries, all equipment was unpacked, and the robotic arms were draped before the patient arrived at the OR room. Afterward, there was a work pause for the scrub nurse while the patient went under anesthesia with the circulating nurse helping with the positioning of the patient followed by the scrub nurse preparing the surgical field. For the next 15 surgeries, there was an uneven pattern where the robotic nurse team sometimes followed the above-described work pattern and at other times, the scrub nurse began to drape the robotic arms while the patient went under anesthesia. This change in pattern was regardless of the team composition. After 20 surgeries, the pattern became more consistent with the scrub nurse and circulating nurse working simultaneously. Technical errors occurred during four surgeries in the preoperative phase without an increase in the preoperative time. If an error occurred during the starting of the system, no other tasks were performed before the technical error was corrected. Over time, the nurses left the OR less often. After the tenth surgery, the nurses began to leave the OR after starting the system to attend their weekly educational sessions. The number of times the robotic nurse team left the OR was not influenced by the move of the robotic system. Whenever a new nurse was introduced to the team, they received bedside training as a supervised circulating nurse. The preoperative and postoperative times were not influenced by this supervision nor when the nurses performed their first independent surgery as either scrub nurses or circulating nurses.

In the perioperative phase, the scrub nurse mainly assisted with instrument change, rinsing the camera lens, and interpreting the technical errors. Rinsing of the camera lens occurred most often at the beginning of the procedure and with instrument change. When technical errors were reported, the scrub nurse had several more instrument changes and rinsing of the camera lens than in surgeries without technical errors. If no technical errors occurred, most often the scrub nurse only had to change the instrument one or two times and none or a few rinsing of the camera were performed during the entire perioperative phase.

In the postoperative phase, the nurses always performed the two tasks simultaneously with the scrub nurse preparing the patient for awakening and the circulating nurse beginning the equipment clean up. The task of equipment cleanup began when the surgeon had undocked the system. When the tasks were finished, the scrub nurse would leave the room, leaving the circulating nurse to help the anesthetics team with the awakening of the patient and the nurse stayed until the patient left the OR. There was a median 28 min gap between finishing the postoperative tasks and the patient leaving the OR.

## Discussion

We described the work pattern and learning curves for an experienced robotic nurse team transferring to a new robotic system. We found the preoperative time did not have a specific learning curve plateau after 30 RARPs but the work pattern seemed to stabilize after 20 RARPs with a multitasking approach between the scrub nurse and circulating nurse. This is in concordance with previous research, which found a significant decrease in preoperative time after 30 RARPS when a consistent surgical team transferred from laparoscopy to robotic surgery [13]. The decrease in preoperative time is important as this is often the leading factor in prolonging operative times in robotic surgery [14, 15]. As in previous studies, we found a prolongation of perioperative time using the Hugo^™^ compared with the Davinci^®^ [16, 17] all in connection with the robotic system itself or unpacking of the sterile gear. Surprisingly, the positioning of the patient was faster for the Hugo^™^ even though there should be no difference in this step between the systems.

The dynamics of the OR are complex and rely on collaboration between multiple groups working together to provide safe and efficient patient care. Most errors in the OR occur outside of the operative field in inexperienced teams due to unclear communication, distractions, excessive workload, fatigue, etc. Experienced teamwork is crucial to reduce the number of adverse events observed in the OR [2, 18–20]. With a more consistent robotic nurse team in our study, perhaps, we could have reduced the preoperative time [13, 21, 22] but this was not possible due to personnel changes, illness, etc. We had multiple team compositions and bedside training of new nurses. This could influence the work patterns of the robotic nurses as they rarely experienced consistency in the OR, thereby challenging their ability to keep up their skills [21]. The number of nurses and different team compositions were a necessity due to the location of the robotic system. The Hugo^™^ was located at an OR away from the regular OR facility due to the space requirements of the system. This made it more difficult and time consuming for the staff to reach the OR resulting in the lack of breaks for the robotic nurses as there rarely were any available nurses trained in the system to substitute. Not all nurses at the facility were trained in the Hugo^™^ due to a nursing shortage resulting in fewer options to send nurses for training in the new robotic system [23]. Perhaps, if the system was closer to the regular OR facility, this problem could have been solved. It would be easier for the substitute nurse to receive immediate help if any issues would occur. However, the safety of this could be debatable if technical issues occur and the substitute is not trained in handling emergencies of the robotic system.

One approach could be to use a registered nurse first assistant (RNFA) as a bedside assistant instead of a medical doctor [24, 25]. The arm components of the Hugo^™^ take up most of the space around the OR table, leaving little room for the bedside assistant and no room for the scrub nurse to assist during surgery. RNFA is an expanded role of the perioperative nurse who can perform intraoperative interventions under the supervision of a surgeon. It can increase the operating room efficiency, reduce change-over times between patients, and increase the quality of patient care [24–26]. This is a way for the OR nurses to expand their roles as professional nurses and clinical experts [22] and could, perhaps, be a long-term solution to the shortage of OR nursing staff, as it broadens the OR nurses’ competencies. However, this still needs to be investigated.

Our study had some limitations. First, we only assessed an experienced robotic nurse team for one procedure type. This might not translate to a nurse team transferring from laparoscopy or to other robotic teams from other specialties. Second, the location of the robotic system could have degraded the working conditions as it was far away from the regular OR facility. Unfortunately, those are the terms for older facilities where there is limited space for larger robotic systems [8]. Third, we did not assess the mental load of the nurses which has previously been shown to increase with the implementation of new robotic technology [7].

The future of robotic surgery will undoubtedly include multiple robotic systems all with different instruments, set-ups, and technical errors. For the implementation process of new systems to be a success, the OR team needs support from their colleagues. Undoubtedly, there will be a disruption to the normal practice with lower productivity while the OR team is learning to use the new system. This will increase the workload until the learning curve has been reached [22]. The facilities must rethink the role of the OR nurses and consider implementing robotic nurse specialists. They can become experts in the use of different robotic systems, especially if multiple specialties are to share the new robotic systems. The robotic nurse specialist can create an overview of the needs of nursing personnel and solve problems with equipment and instruments faster. The future will most likely require robotic nurses to be proficient with not only one robotic system but in general medical robotics [3, 27].

## Conclusion

We found that the work pattern seemed to stabilize after 20 RARPs but with a continued decrease in preoperative time without a learning curve plateau. The robotic nurse team suffered from few breaks and long working hours because only a few nurses at our facility were trained in the Hugo™ system.

## Supplementary Information

Below is the link to the electronic supplementary material.

Supplementary file1 Fig. S1 All of the Timelines of the work pattern of the robotic nurse team (PDF 167 KB)

Supplementary file 2 File S1 The Strengthening the Reporting of Observational Studies in Epidemiology (STROBE)Statement: guidelines for reporting observational studies (DOC 101 KB)

## Data Availability

Data is available on reasonable request from the authors.
