# Supplementary material for: How experienced robotic nurses adapt to the Hugo™ RAS system
Source: J Robot Surg. 2024 Mar 11;18(1):114. doi: 10.1007/s11701-024-01878-x (PMC10927759; doi:10.1007/s11701-024-01878-x)
Supplement: Supplementary file 1 — Supplementary file1 Fig. S1 All of the Timelines of the work pattern of the robotic nurse team (PDF 167 KB) [file 11701_2024_1878_MOESM1_ESM.pdf]

Supplementary Figure 1 All of the Timelines of the work pattern of the robotic nurse team

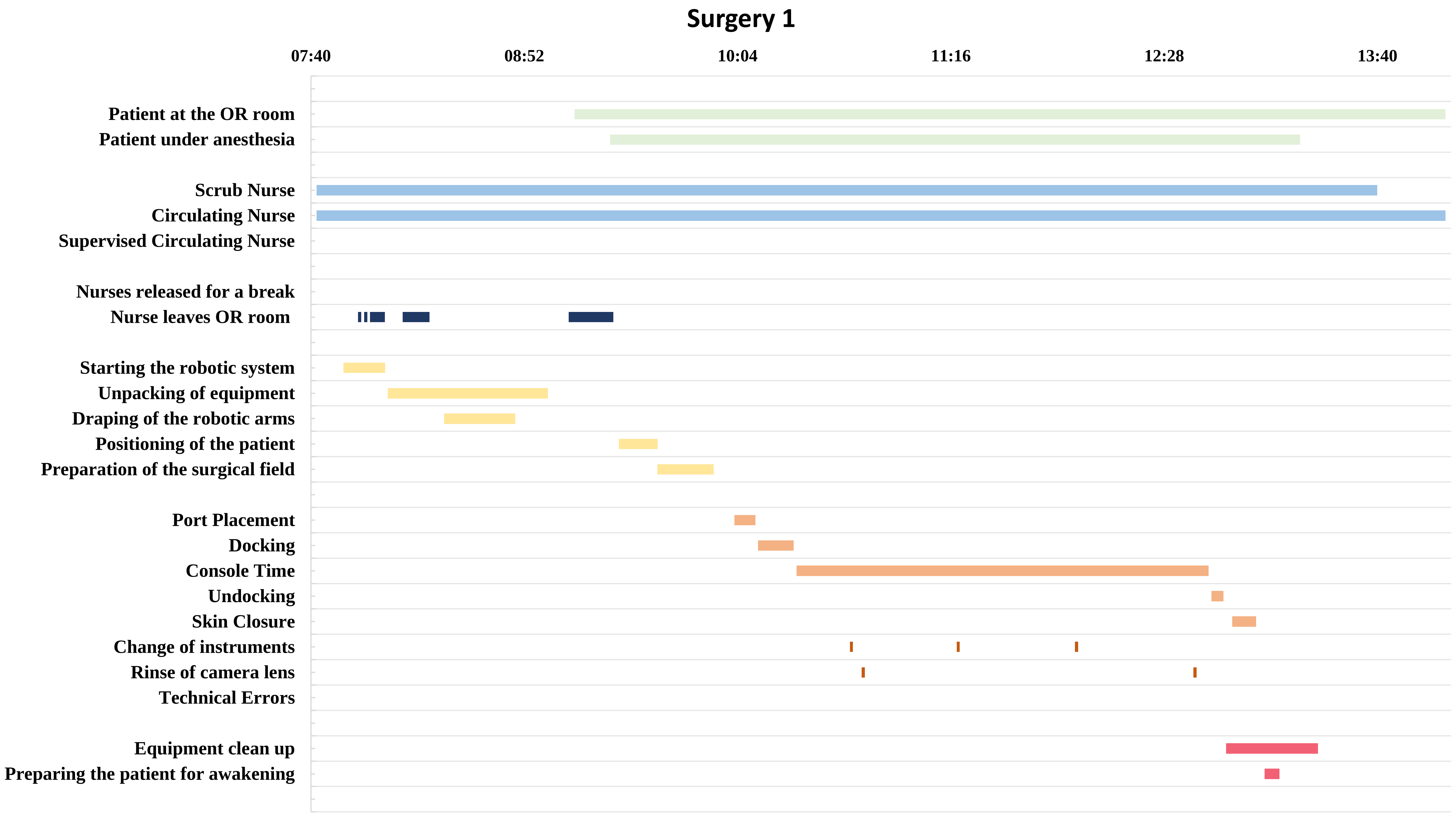

Surgery 1
07:40
08:52
10:04
11:16
12:28
13:40
Patient at the OR room
Patient under anesthesia
Scrub Nurse
Circulating Nurse
Supervised Circulating Nurse
Nurses released for a break
Nurse leaves OR room
Starting the robotic system
Unpacking of equipment
Draping of the robotic arms
Positioning of the patient
Preparation of the surgical field
Port Placement
Docking
Console Time
Undocking
Skin Closure
Change of instruments
Rinse of camera lens
Technical Errors
Equipment clean up
Preparing the patient for awakening

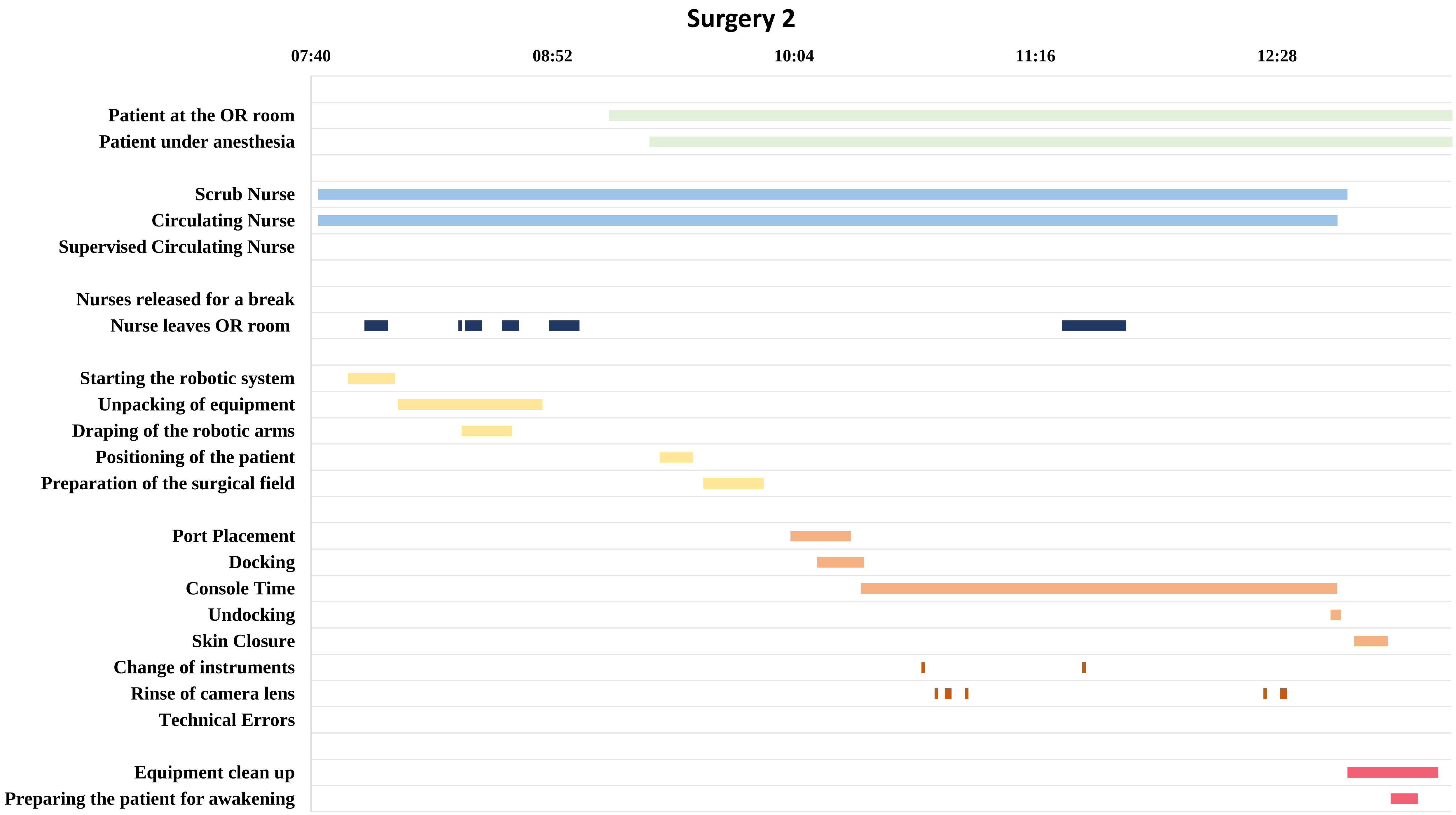

Surgery 2
07:40
08:52
10:04
11:16
12:28
Patient at the OR room
Patient under anesthesia
Scrub Nurse
Circulating Nurse
Supervised Circulating Nurse
Nurses released for a break
Nurse leaves OR room
Starting the robotic system
Unpacking of equipment
Draping of the robotic arms
Positioning of the patient
Preparation of the surgical field
Port Placement
Docking
Console Time
Undocking
Skin Closure
Change of instruments
Rinse of camera lens
Technical Errors
Equipment clean up
Preparing the patient for awakening

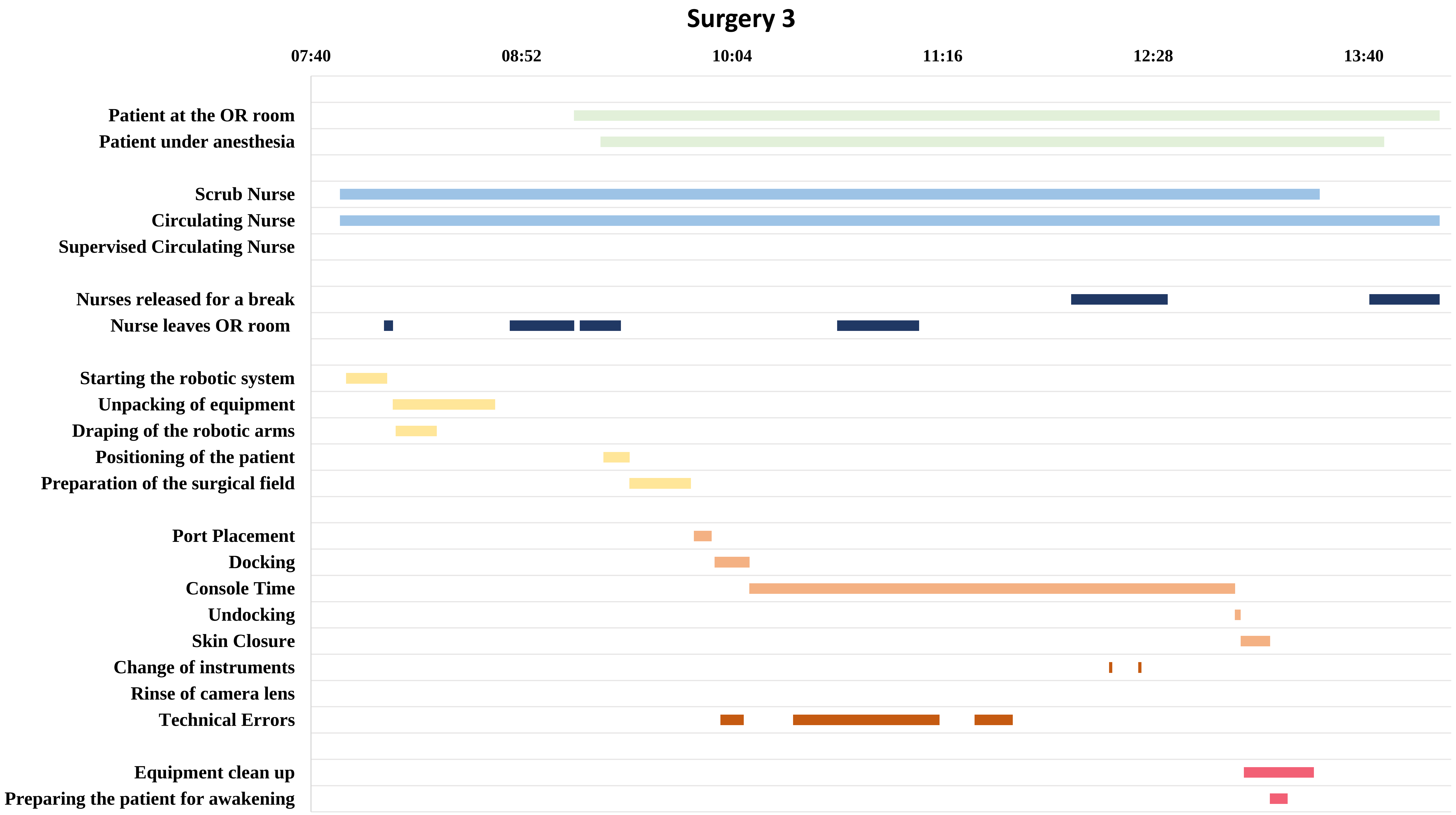

Surgery 3
07:40
08:52
10:04
11:16
12:28
13:40
Patient at the OR room
Patient under anesthesia
Scrub Nurse
Circulating Nurse
Supervised Circulating Nurse
Nurses released for a break
Nurse leaves OR room
Starting the robotic system
Unpacking of equipment
Draping of the robotic arms
Positioning of the patient
Preparation of the surgical field
Port Placement
Docking
Console Time
Undocking
Skin Closure
Change of instruments
Rinse of camera lens
Technical Errors
Equipment clean up
Preparing the patient for awakening

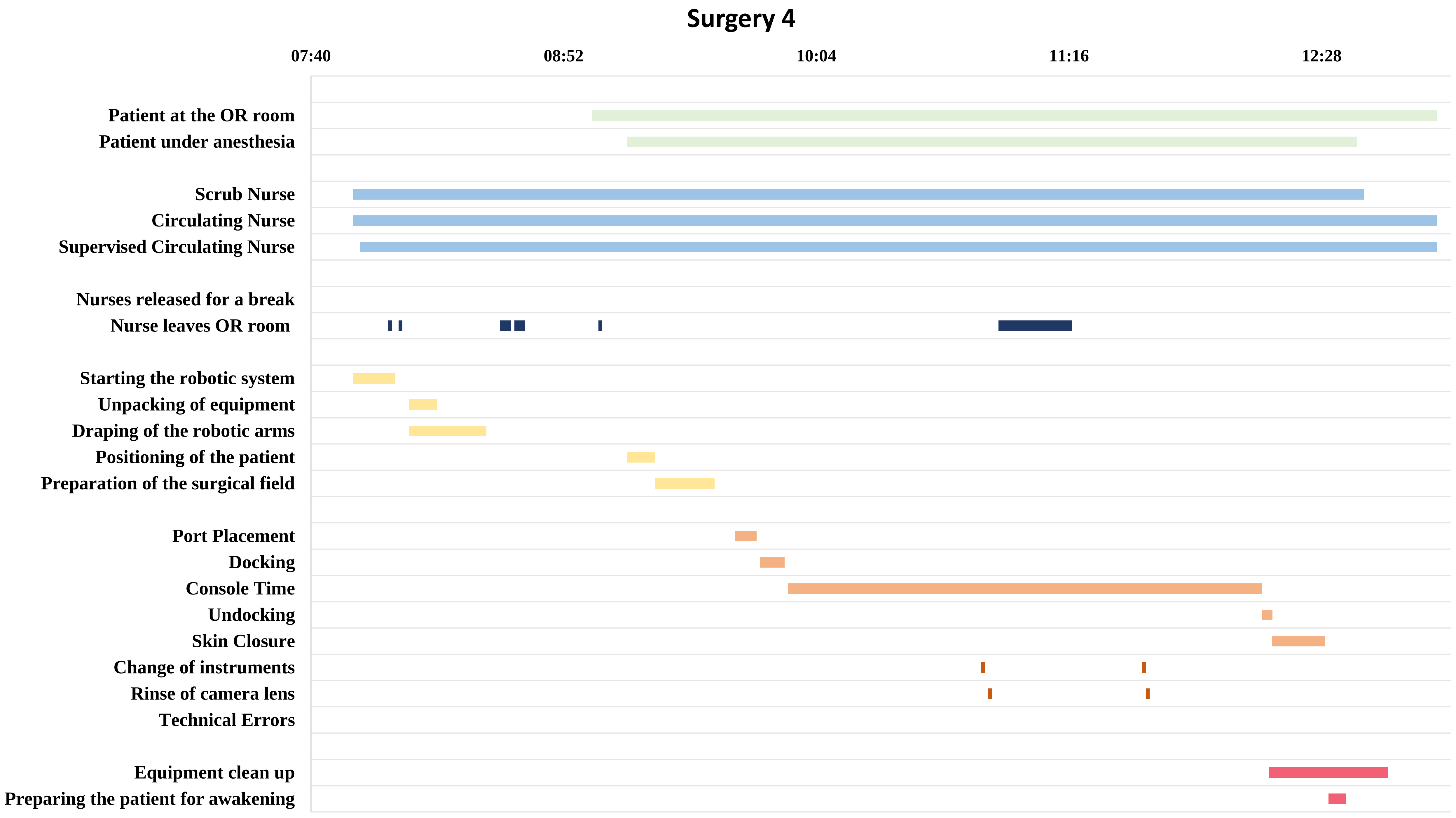
Surgery 4
07:40
08:52
10:04
11:16
12:28
Patient at the OR room
Patient under anesthesia
Scrub Nurse
Circulating Nurse
Supervised Circulating Nurse
Nurses released for a break
Nurse leaves OR room
Starting the robotic system
Unpacking of equipment
Draping of the robotic arms
Positioning of the patient
Preparation of the surgical field
Port Placement
Docking
Console Time
Undocking
Skin Closure
Change of instruments
Rinse of camera lens
Technical Errors
Equipment clean up
Preparing the patient for awakening

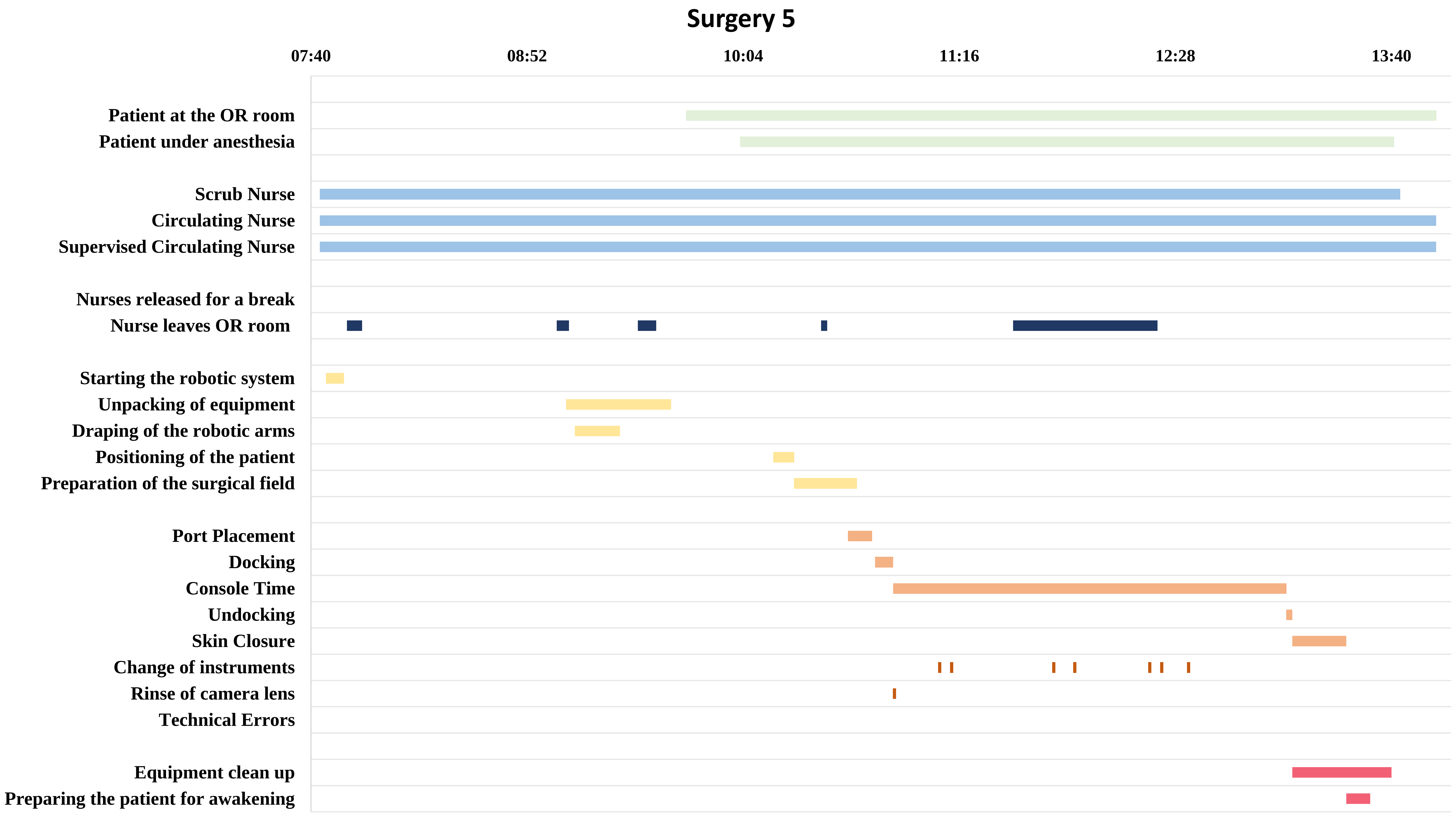
Surgery 5
07:40
08:52
10:04
11:16
12:28
13:40
Patient at the OR room
Patient under anesthesia
Scrub Nurse
Circulating Nurse
Supervised Circulating Nurse
Nurses released for a break
Nurse leaves OR room
Starting the robotic system
Unpacking of equipment
Draping of the robotic arms
Positioning of the patient
Preparation of the surgical field
Port Placement
Docking
Console Time
Undocking
Skin Closure
Change of instruments
Rinse of camera lens
Technical Errors
Equipment clean up
Preparing the patient for awakening

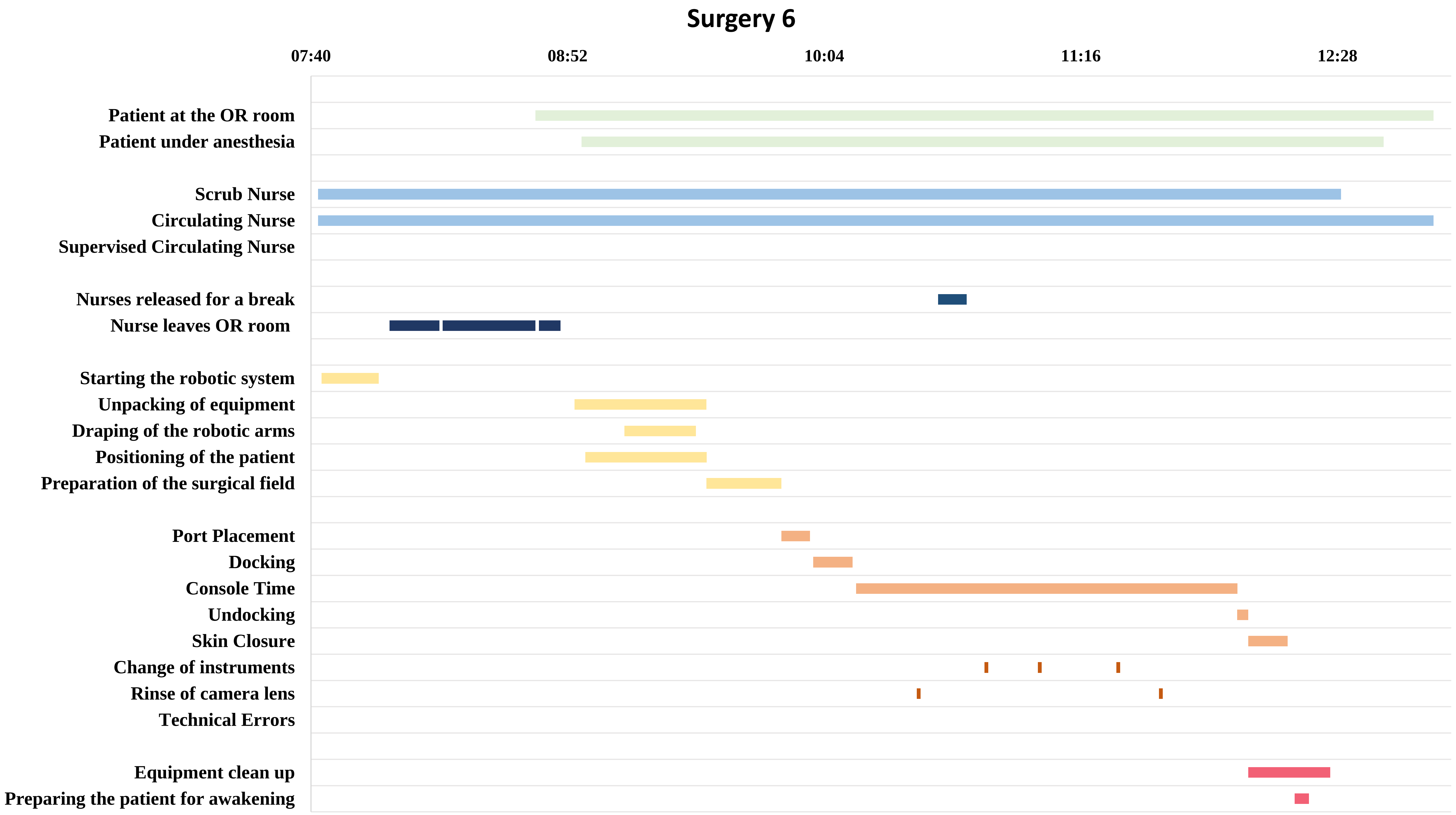
Surgery 6
07:40
08:52
10:04
11:16
12:28
Patient at the OR room
Patient under anesthesia
Scrub Nurse
Circulating Nurse
Supervised Circulating Nurse
Nurses released for a break
Nurse leaves OR room
Starting the robotic system
Unpacking of equipment
Draping of the robotic arms
Positioning of the patient
Preparation of the surgical field
Port Placement
Docking
Console Time
Undocking
Skin Closure
Change of instruments
Rinse of camera lens
Technical Errors
Equipment clean up
Preparing the patient for awakening

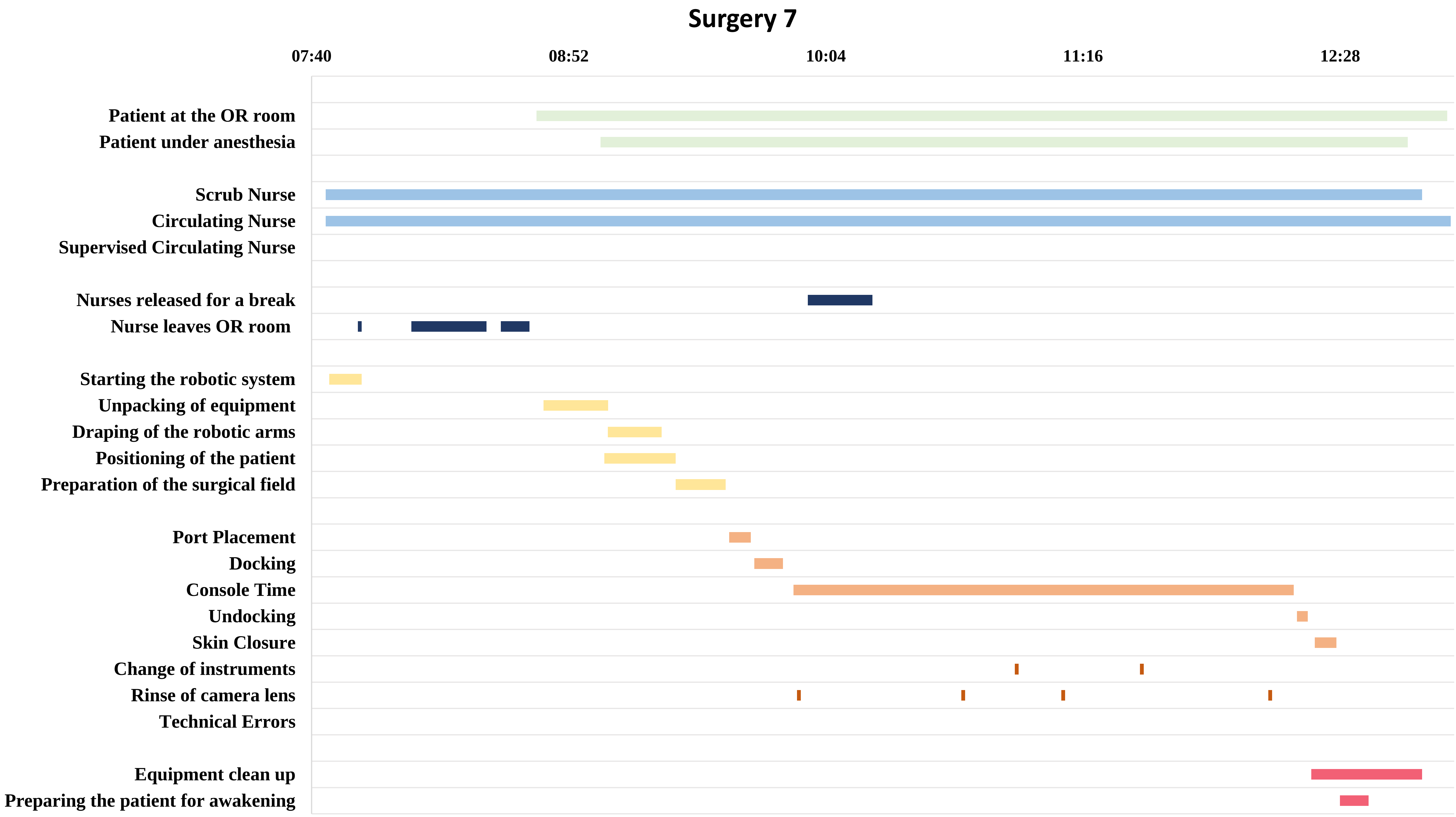
Surgery 7
07:40
08:52
10:04
11:16
12:28
Patient at the OR room
Patient under anesthesia
Scrub Nurse
Circulating Nurse
Supervised Circulating Nurse
Nurses released for a break
Nurse leaves OR room
Starting the robotic system
Unpacking of equipment
Draping of the robotic arms
Positioning of the patient
Preparation of the surgical field
Port Placement
Docking
Console Time
Undocking
Skin Closure
Change of instruments
Rinse of camera lens
Technical Errors
Equipment clean up
Preparing the patient for awakening

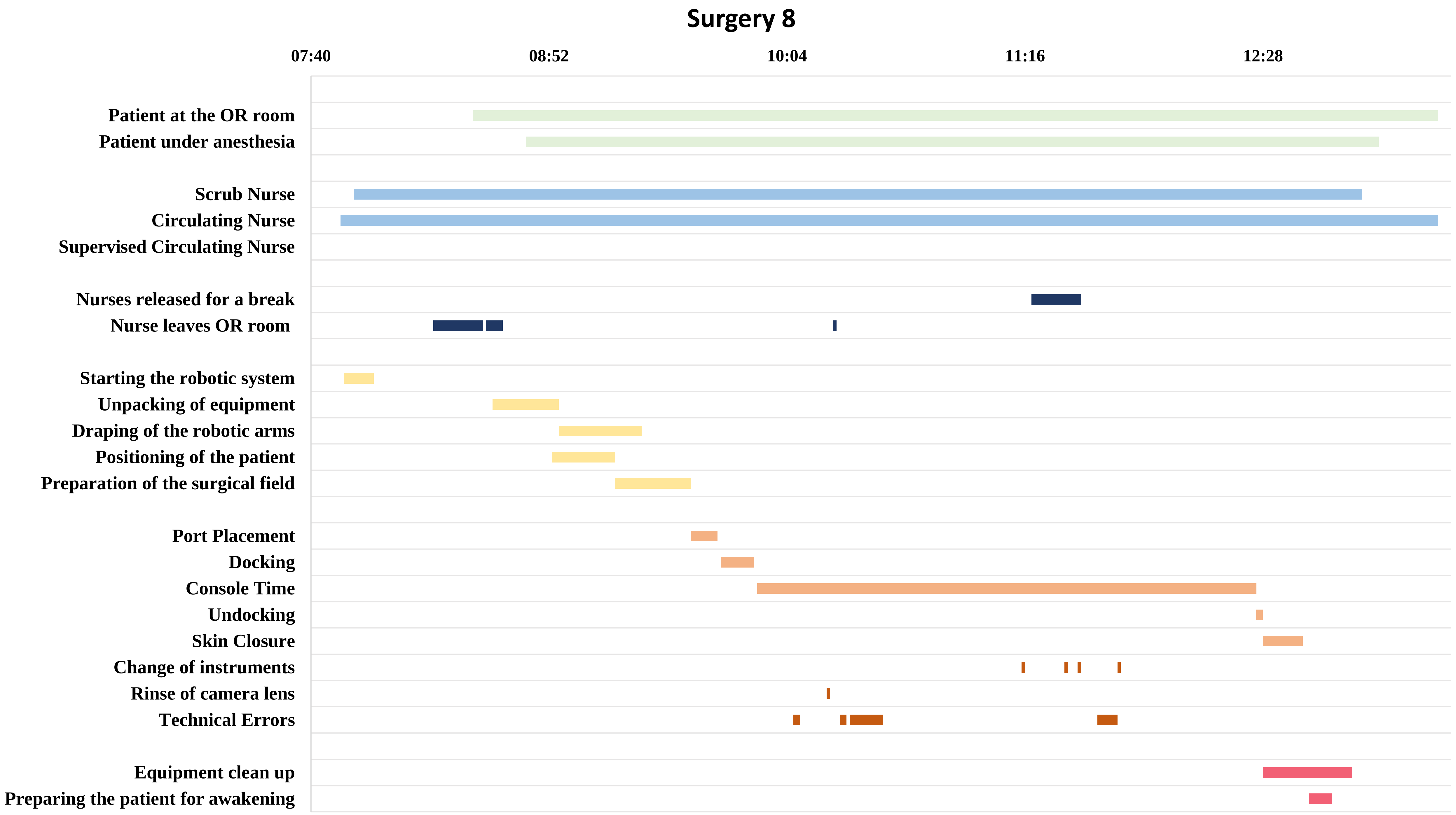
Surgery 8
07:40
08:52
10:04
11:16
12:28
Patient at the OR room
Patient under anesthesia
Scrub Nurse
Circulating Nurse
Supervised Circulating Nurse
Nurses released for a break
Nurse leaves OR room
Starting the robotic system
Unpacking of equipment
Draping of the robotic arms
Positioning of the patient
Preparation of the surgical field
Port Placement
Docking
Console Time
Undocking
Skin Closure
Change of instruments
Rinse of camera lens
Technical Errors
Equipment clean up
Preparing the patient for awakening

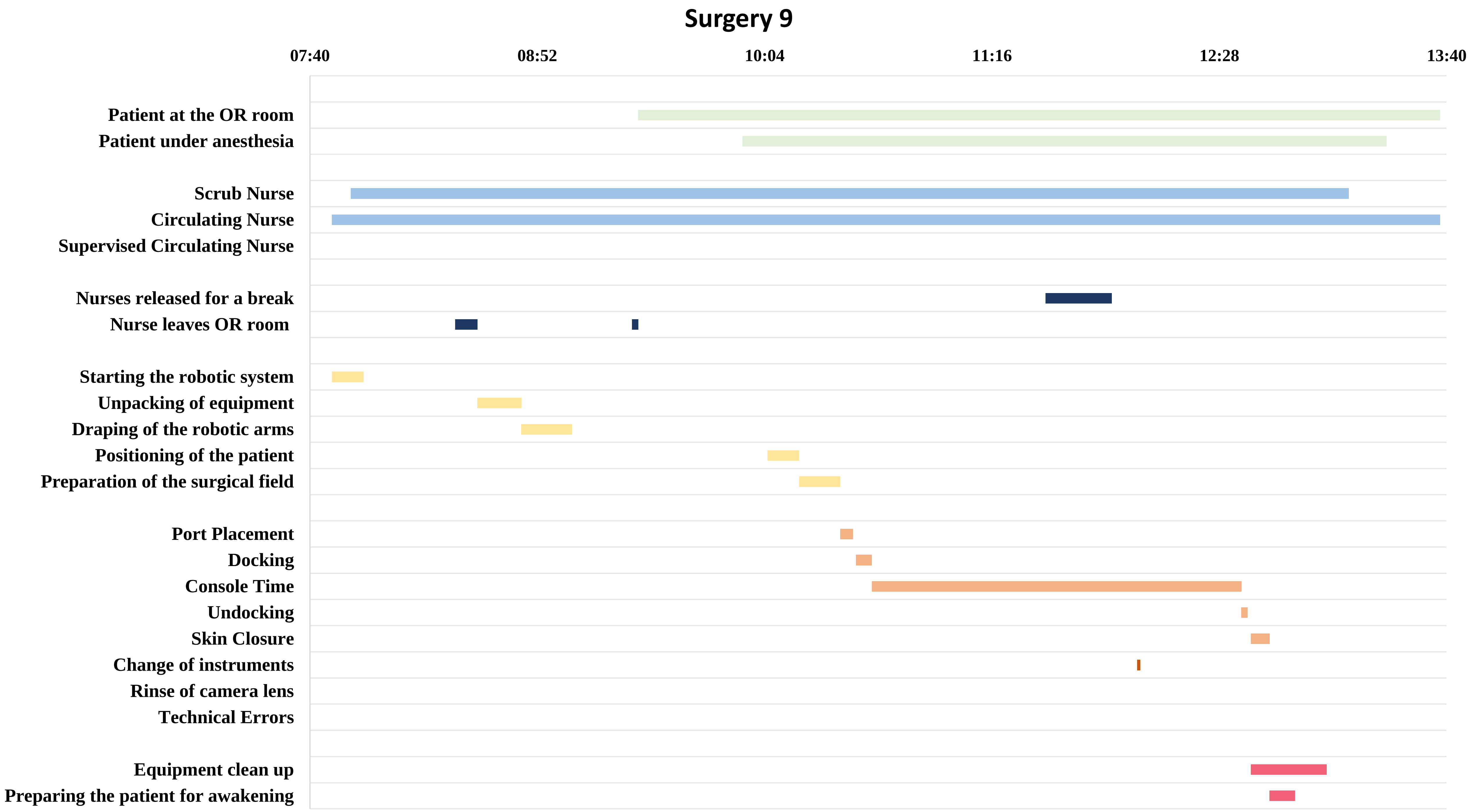

Surgery 9
07:40
08:52
10:04
11:16
12:28
13:40
Patient at the OR room
Patient under anesthesia
Scrub Nurse
Circulating Nurse
Supervised Circulating Nurse
Nurses released for a break
Nurse leaves OR room
Starting the robotic system
Unpacking of equipment
Draping of the robotic arms
Positioning of the patient
Preparation of the surgical field
Port Placement
Docking
Console Time
Undocking
Skin Closure
Change of instruments
Rinse of camera lens
Technical Errors
Equipment clean up
Preparing the patient for awakening

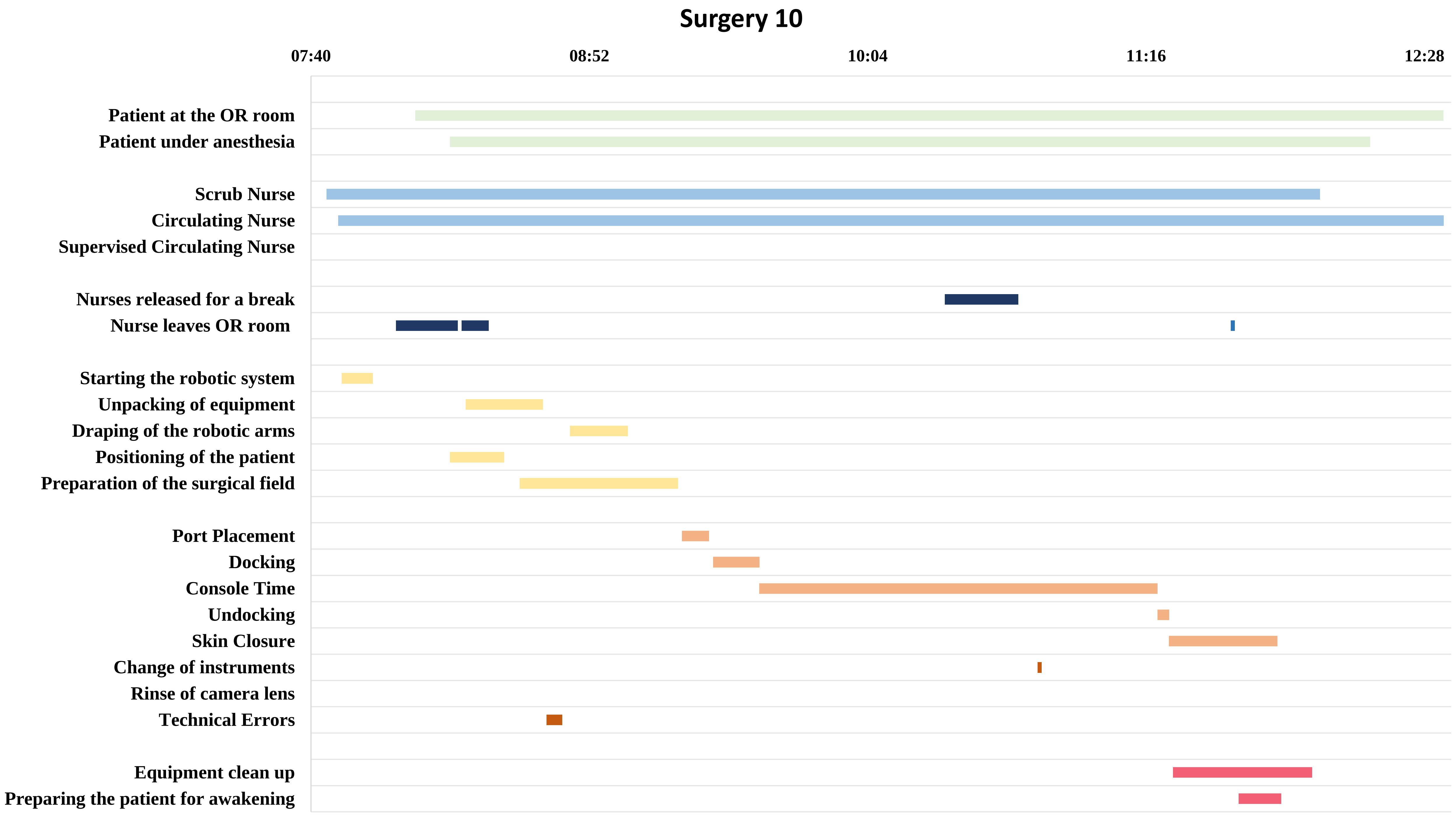
Surgery 10
07:40
08:52
10:04
11:16
12:28
Patient at the OR room
Patient under anesthesia
Scrub Nurse
Circulating Nurse
Supervised Circulating Nurse
Nurses released for a break
Nurse leaves OR room
Starting the robotic system
Unpacking of equipment
Draping of the robotic arms
Positioning of the patient
Preparation of the surgical field
Port Placement
Docking
Console Time
Undocking
Skin Closure
Change of instruments
Rinse of camera lens
Technical Errors
Equipment clean up
Preparing the patient for awakening

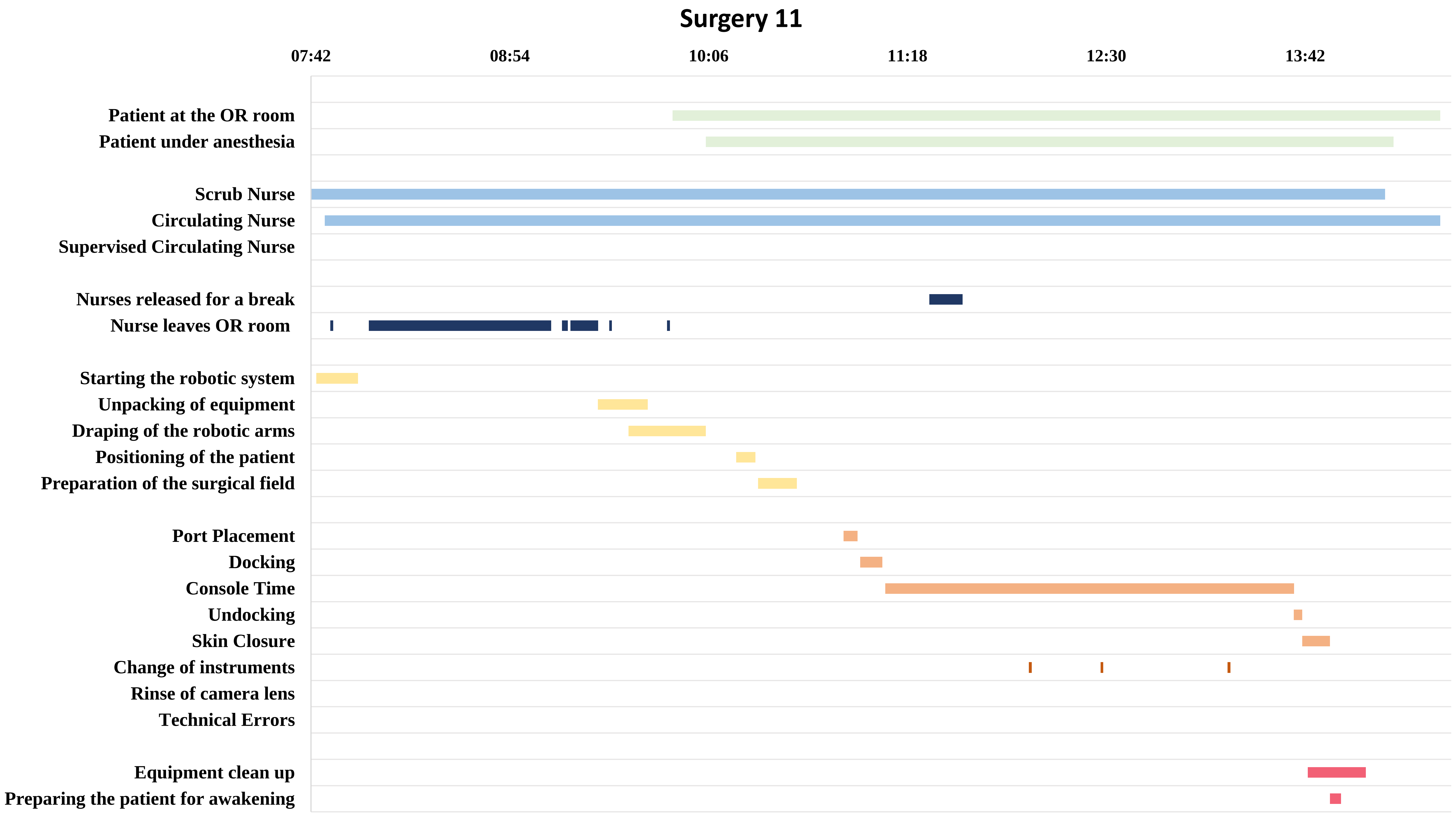
Surgery 11
07:42
08:54
10:06
11:18
12:30
13:42
Patient at the OR room
Patient under anesthesia
Scrub Nurse
Circulating Nurse
Supervised Circulating Nurse
Nurses released for a break
Nurse leaves OR room
Starting the robotic system
Unpacking of equipment
Draping of the robotic arms
Positioning of the patient
Preparation of the surgical field
Port Placement
Docking
Console Time
Undocking
Skin Closure
Change of instruments
Rinse of camera lens
Technical Errors
Equipment clean up
Preparing the patient for awakening

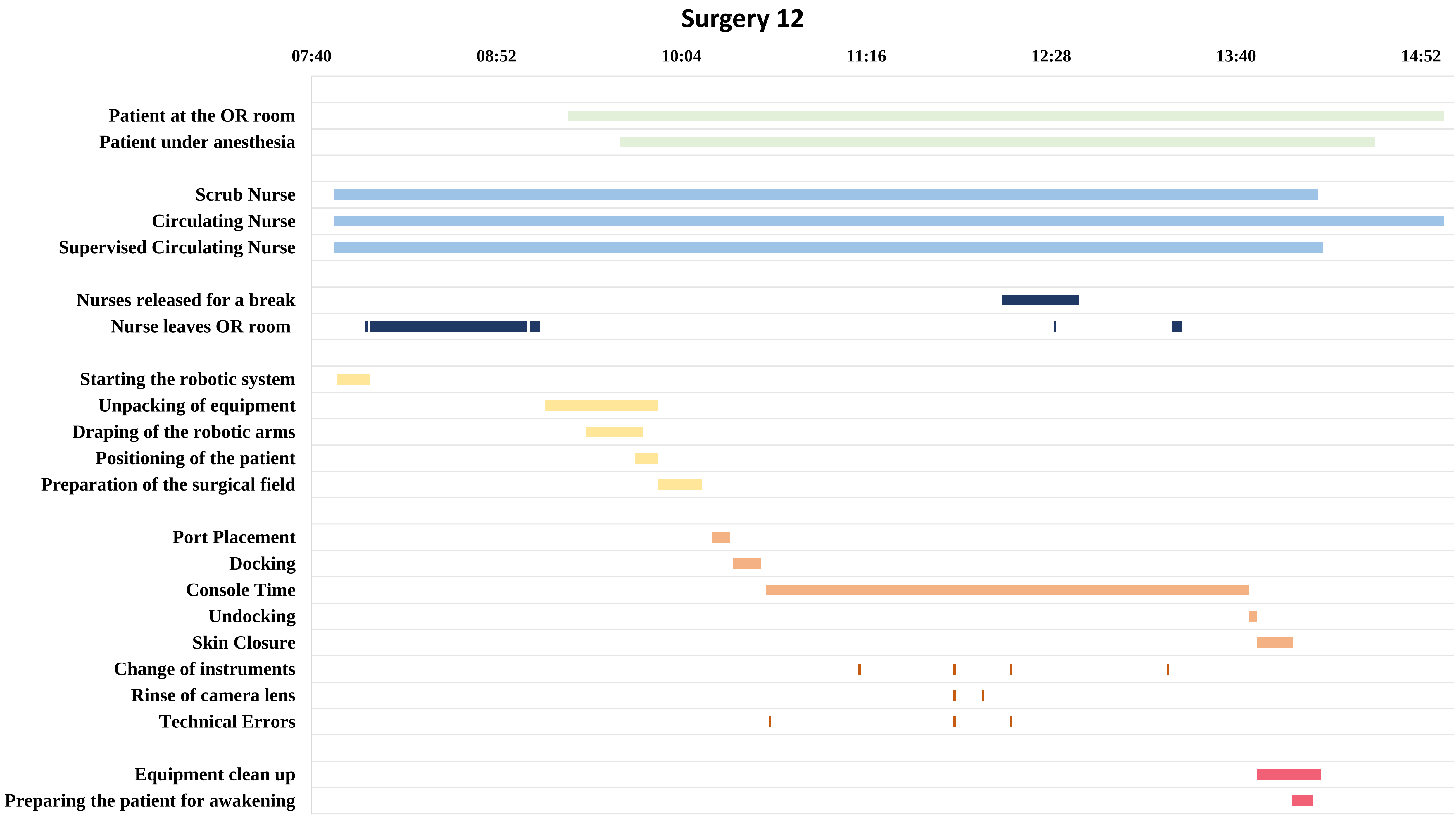

Surgery 12
07:40
08:52
10:04
11:16
12:28
13:40
14:52
Patient at the OR room
Patient under anesthesia
Scrub Nurse
Circulating Nurse
Supervised Circulating Nurse
Nurses released for a break
Nurse leaves OR room
Starting the robotic system
Unpacking of equipment
Draping of the robotic arms
Positioning of the patient
Preparation of the surgical field
Port Placement
Docking
Console Time
Undocking
Skin Closure
Change of instruments
Rinse of camera lens
Technical Errors
Equipment clean up
Preparing the patient for awakening

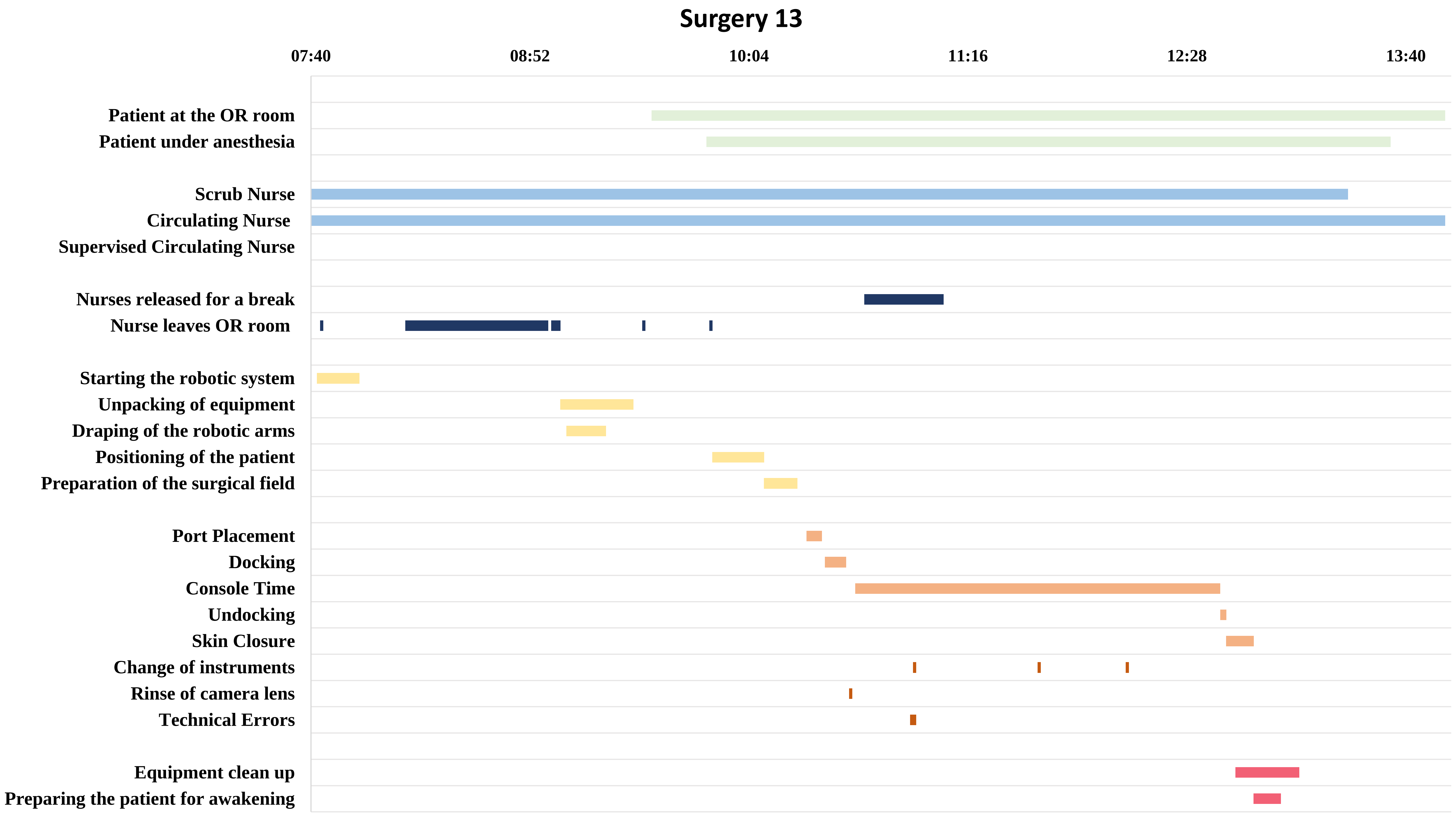
Surgery 13
07:40
08:52
10:04
11:16
12:28
13:40
Patient at the OR room
Patient under anesthesia
Scrub Nurse
Circulating Nurse
Supervised Circulating Nurse
Nurses released for a break
Nurse leaves OR room
Starting the robotic system
Unpacking of equipment
Draping of the robotic arms
Positioning of the patient
Preparation of the surgical field
Port Placement
Docking
Console Time
Undocking
Skin Closure
Change of instruments
Rinse of camera lens
Technical Errors
Equipment clean up
Preparing the patient for awakening

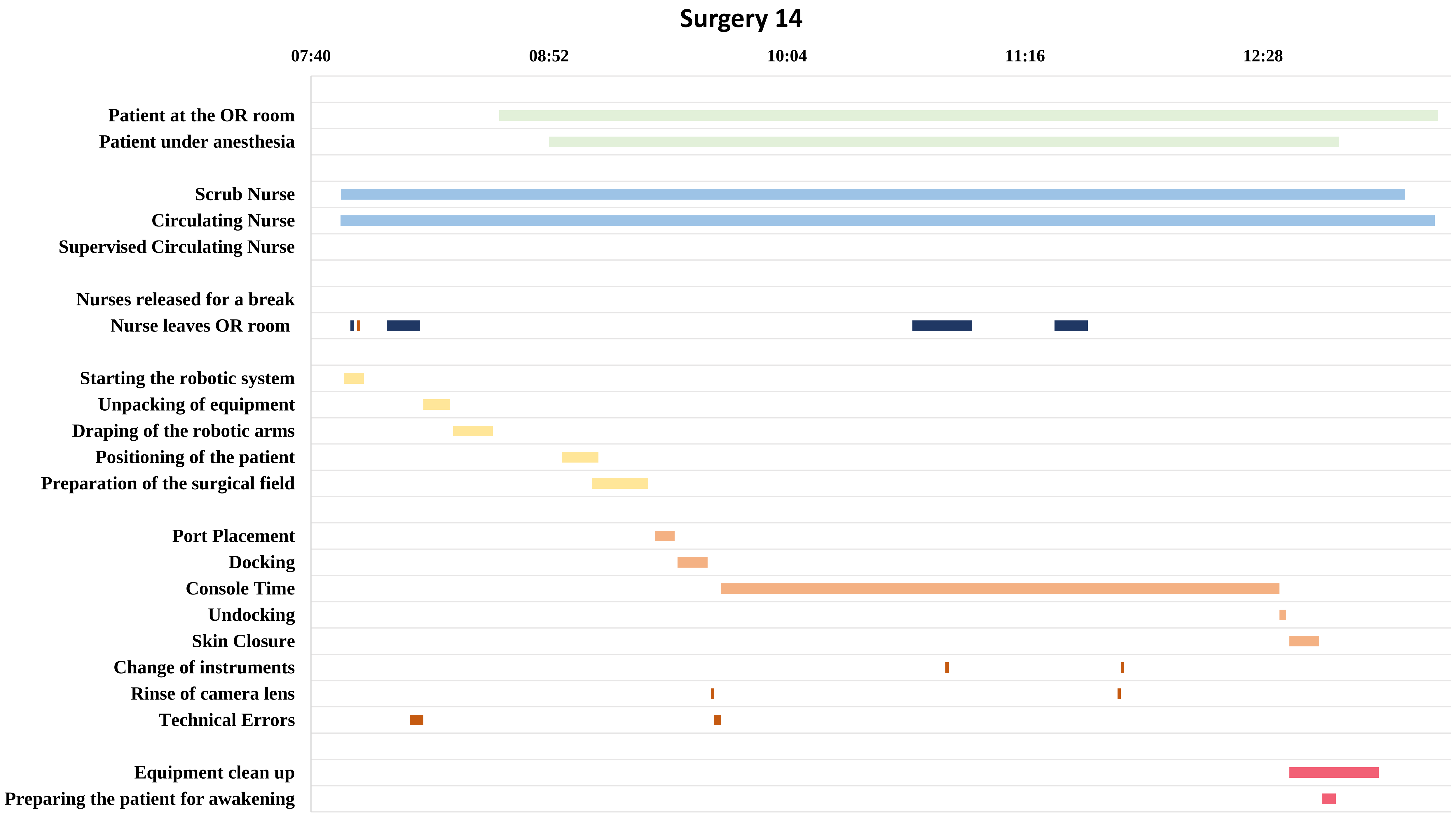

Surgery 14
07:40
08:52
10:04
11:16
12:28
Patient at the OR room
Patient under anesthesia
Scrub Nurse
Circulating Nurse
Supervised Circulating Nurse
Nurses released for a break
Nurse leaves OR room
Starting the robotic system
Unpacking of equipment
Draping of the robotic arms
Positioning of the patient
Preparation of the surgical field
Port Placement
Docking
Console Time
Undocking
Skin Closure
Change of instruments
Rinse of camera lens
Technical Errors
Equipment clean up
Preparing the patient for awakening

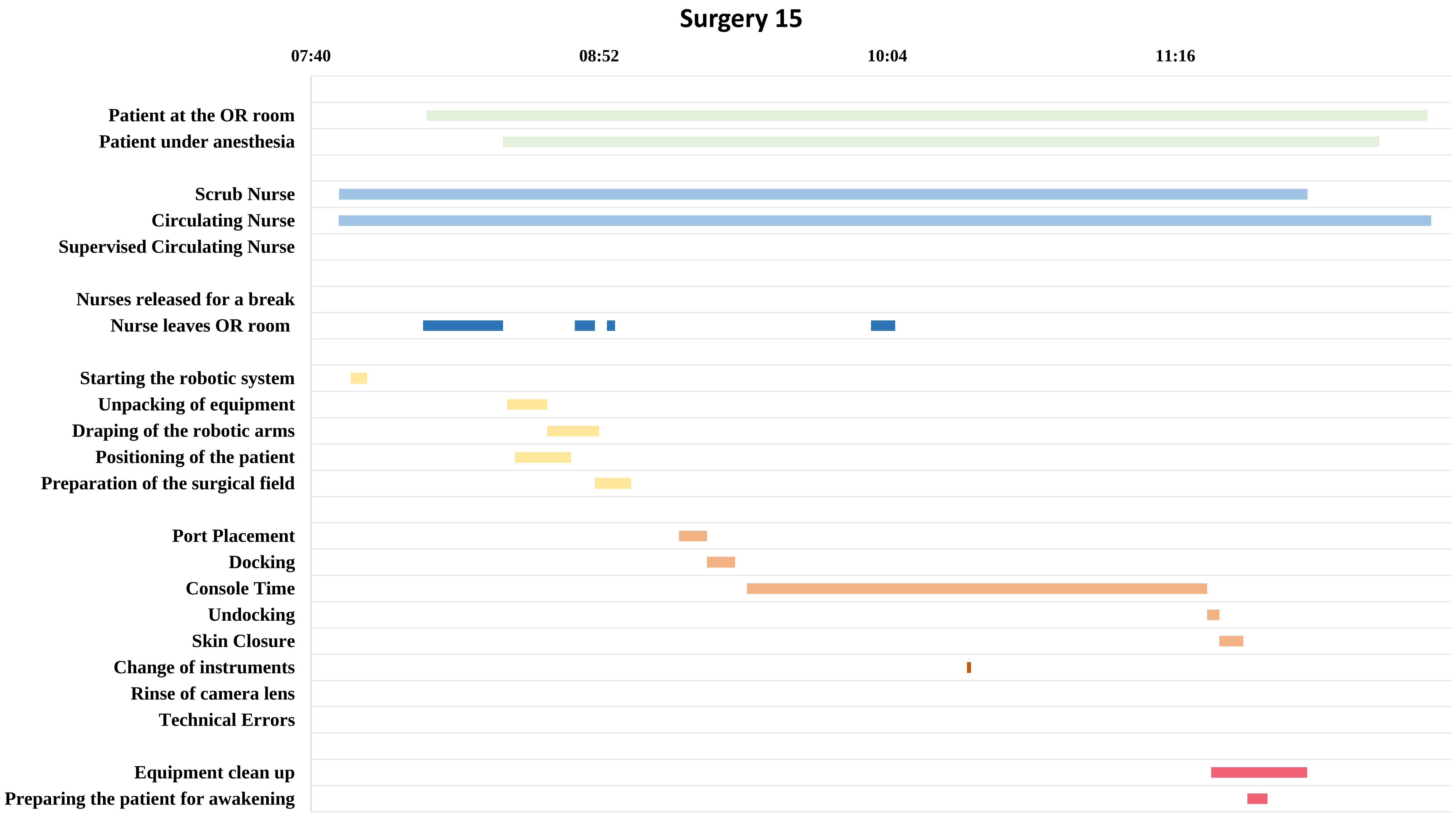
Surgery 15
07:40
08:52
10:04
11:16
Patient at the OR room
Patient under anesthesia
Scrub Nurse
Circulating Nurse
Supervised Circulating Nurse
Nurses released for a break
Nurse leaves OR room
Starting the robotic system
Unpacking of equipment
Draping of the robotic arms
Positioning of the patient
Preparation of the surgical field
Port Placement
Docking
Console Time
Undocking
Skin Closure
Change of instruments
Rinse of camera lens
Technical Errors
Equipment clean up
Preparing the patient for awakening

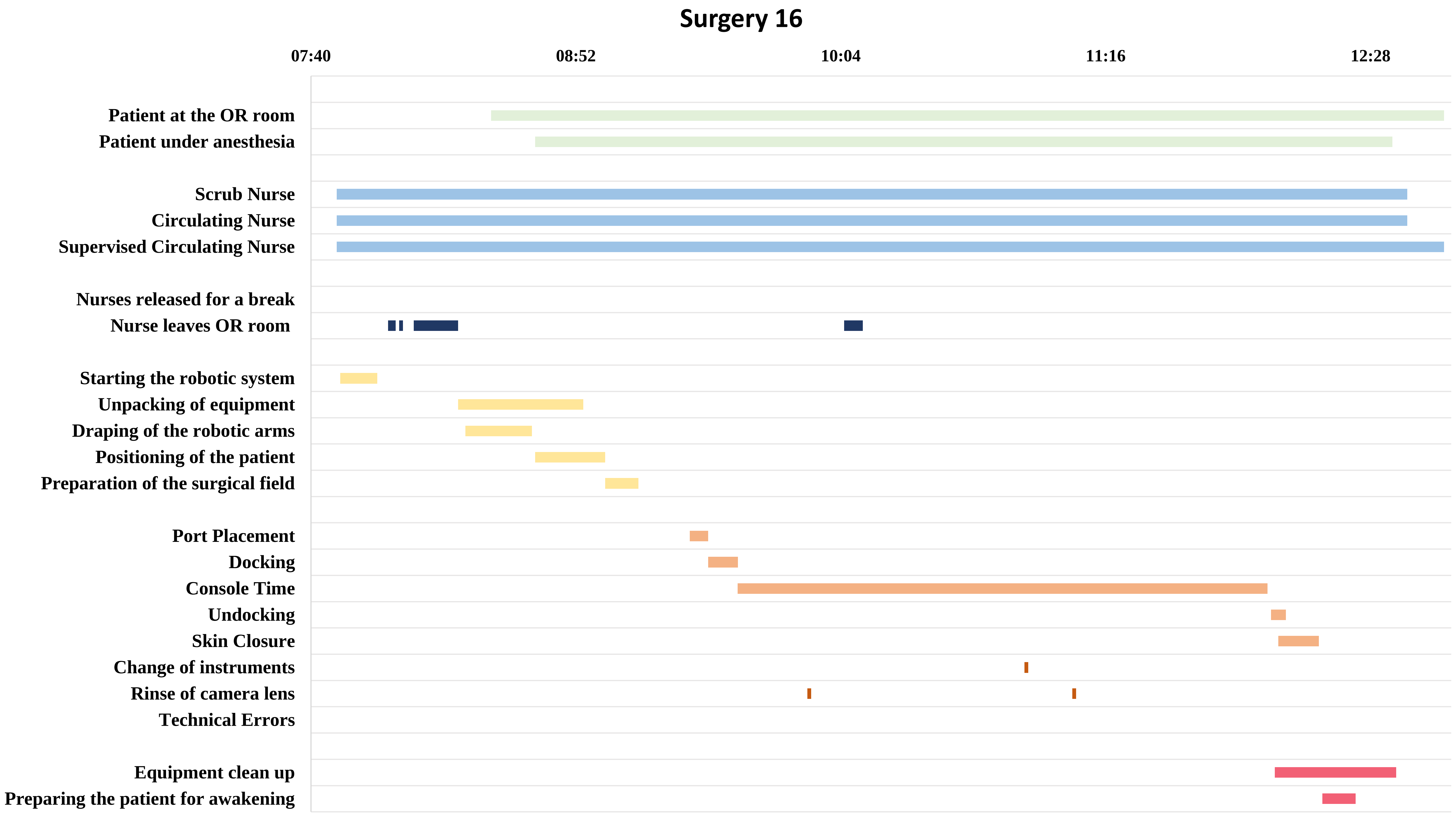
Surgery 16
07:40
08:52
10:04
11:16
12:28
Patient at the OR room
Patient under anesthesia
Scrub Nurse
Circulating Nurse
Supervised Circulating Nurse
Nurses released for a break
Nurse leaves OR room
Starting the robotic system
Unpacking of equipment
Draping of the robotic arms
Positioning of the patient
Preparation of the surgical field
Port Placement
Docking
Console Time
Undocking
Skin Closure
Change of instruments
Rinse of camera lens
Technical Errors
Equipment clean up
Preparing the patient for awakening

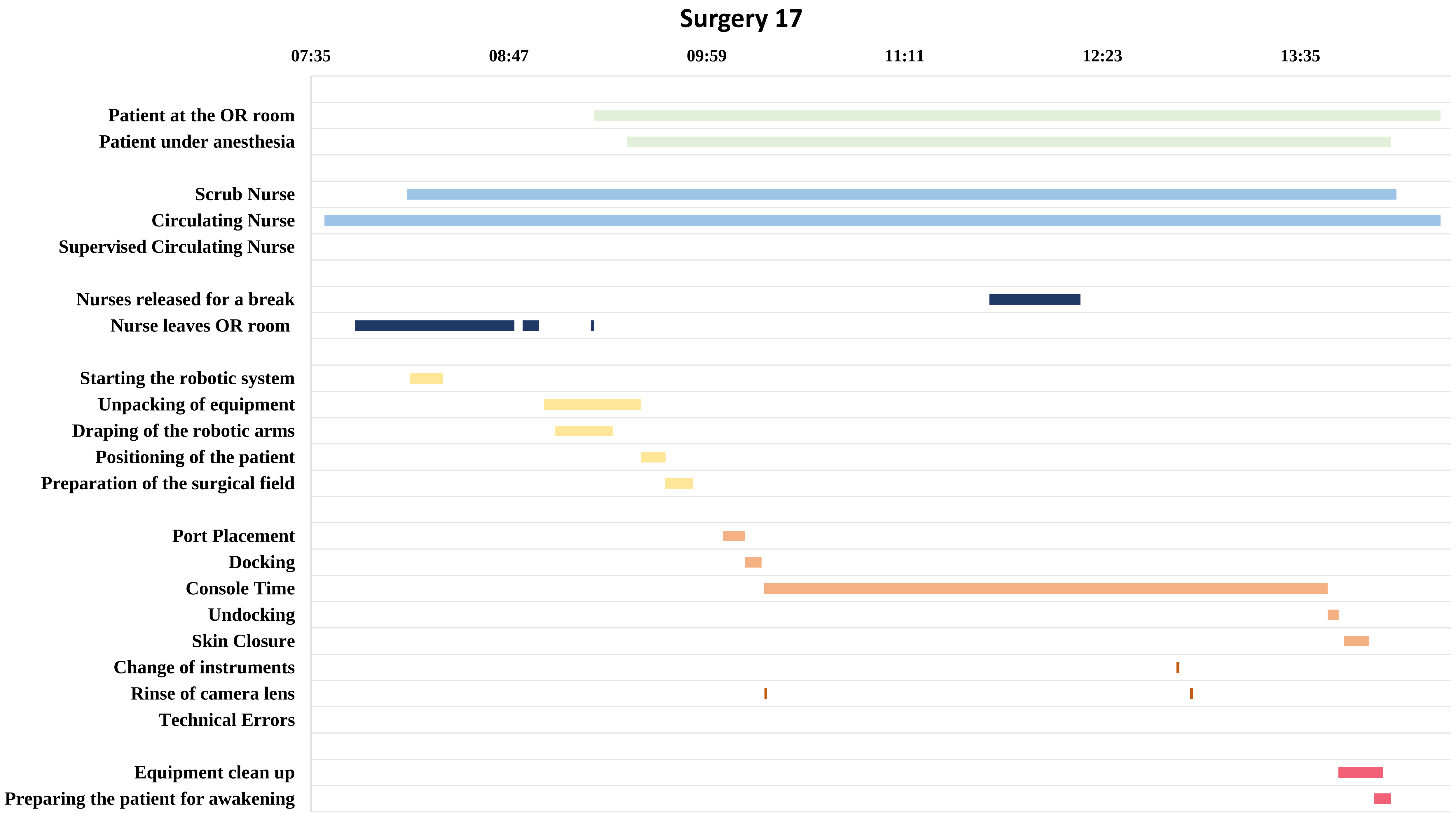
Surgery 17
07:35
08:47
09:59
11:11
12:23
13:35
Patient at the OR room
Patient under anesthesia
Scrub Nurse
Circulating Nurse
Supervised Circulating Nurse
Nurses released for a break
Nurse leaves OR room
Starting the robotic system
Unpacking of equipment
Draping of the robotic arms
Positioning of the patient
Preparation of the surgical field
Port Placement
Docking
Console Time
Undocking
Skin Closure
Change of instruments
Rinse of camera lens
Technical Errors
Equipment clean up
Preparing the patient for awakening

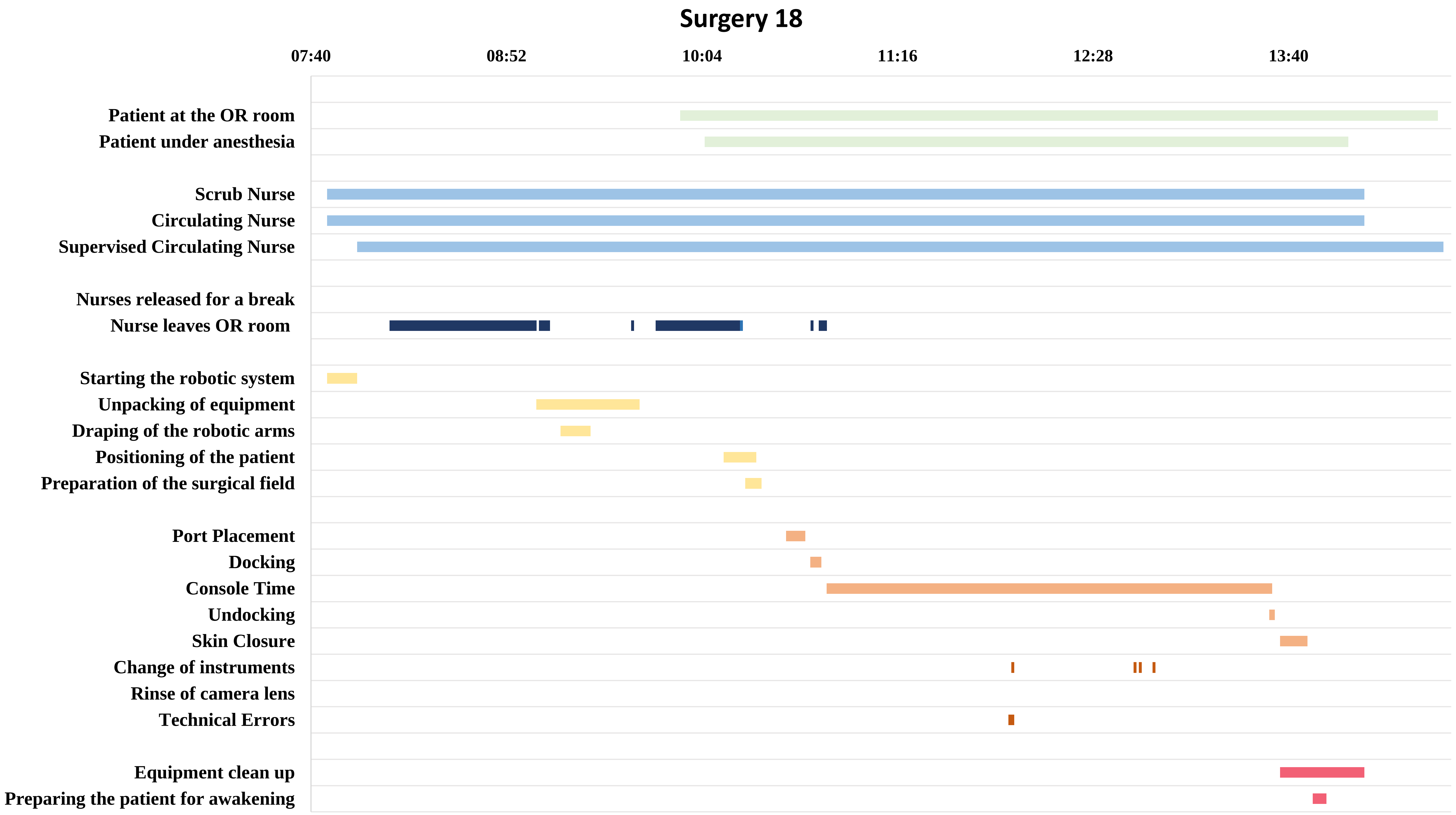
Surgery 18
07:40
08:52
10:04
11:16
12:28
13:40
Patient at the OR room
Patient under anesthesia
Scrub Nurse
Circulating Nurse
Supervised Circulating Nurse
Nurses released for a break
Nurse leaves OR room
Starting the robotic system
Unpacking of equipment
Draping of the robotic arms
Positioning of the patient
Preparation of the surgical field
Port Placement
Docking
Console Time
Undocking
Skin Closure
Change of instruments
Rinse of camera lens
Technical Errors
Equipment clean up
Preparing the patient for awakening

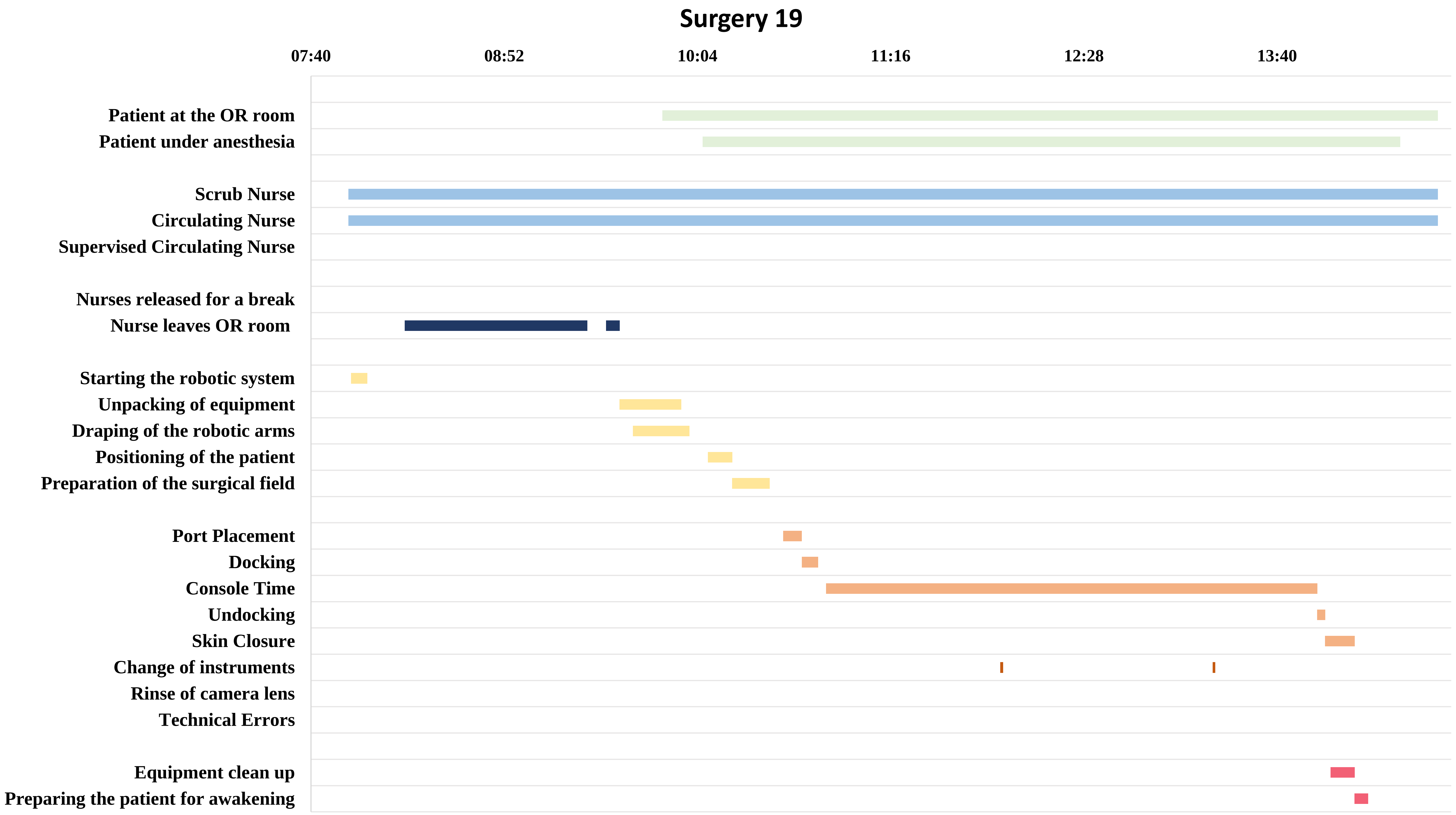
Surgery 19
07:40
08:52
10:04
11:16
12:28
13:40
Patient at the OR room
Patient under anesthesia
Scrub Nurse
Circulating Nurse
Supervised Circulating Nurse
Nurses released for a break
Nurse leaves OR room
Starting the robotic system
Unpacking of equipment
Draping of the robotic arms
Positioning of the patient
Preparation of the surgical field
Port Placement
Docking
Console Time
Undocking
Skin Closure
Change of instruments
Rinse of camera lens
Technical Errors
Equipment clean up
Preparing the patient for awakening

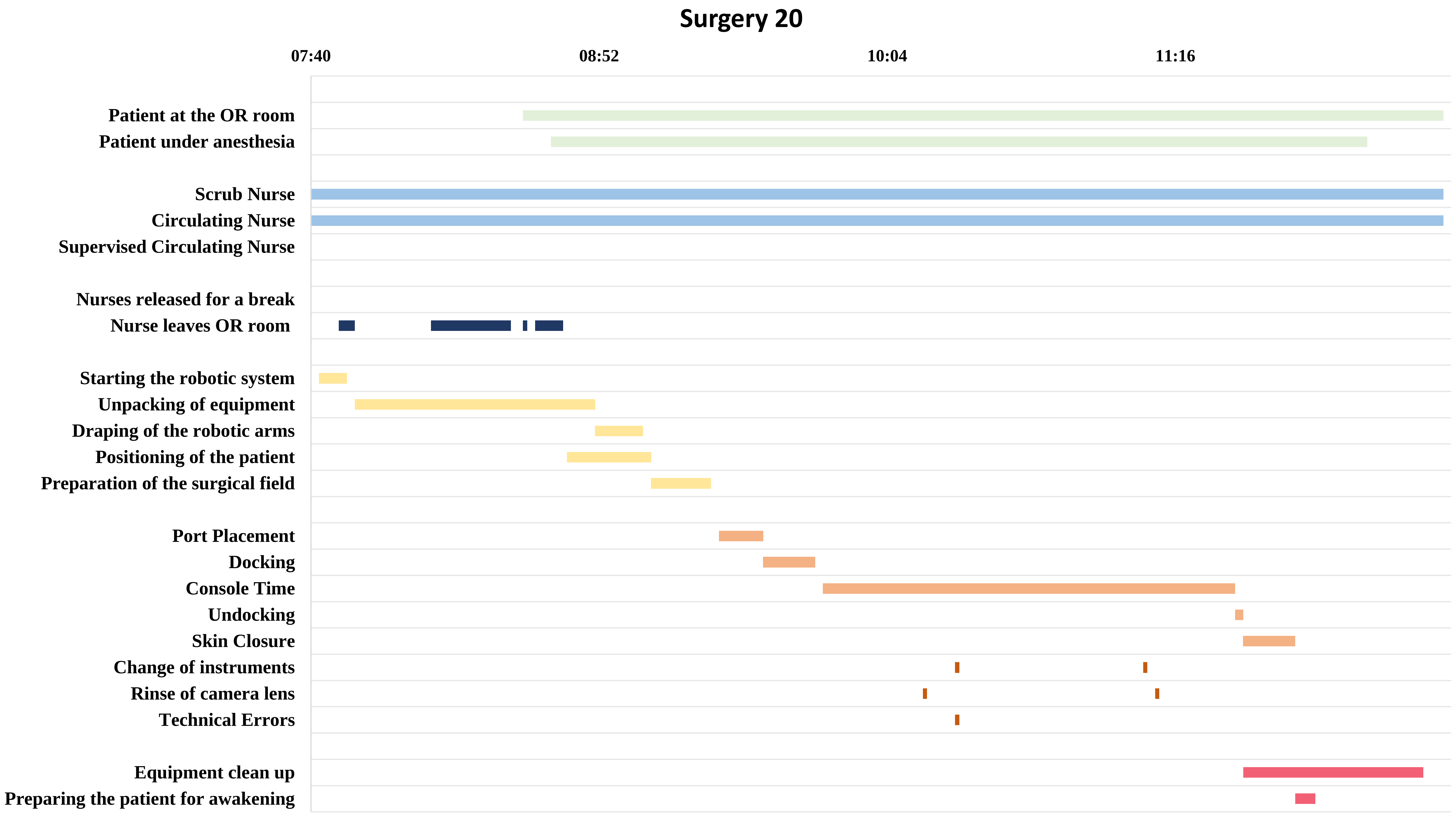
Surgery 20
07:40
08:52
10:04
11:16
Patient at the OR room
Patient under anesthesia
Scrub Nurse
Circulating Nurse
Supervised Circulating Nurse
Nurses released for a break
Nurse leaves OR room
Starting the robotic system
Unpacking of equipment
Draping of the robotic arms
Positioning of the patient
Preparation of the surgical field
Port Placement
Docking
Console Time
Undocking
Skin Closure
Change of instruments
Rinse of camera lens
Technical Errors
Equipment clean up
Preparing the patient for awakening

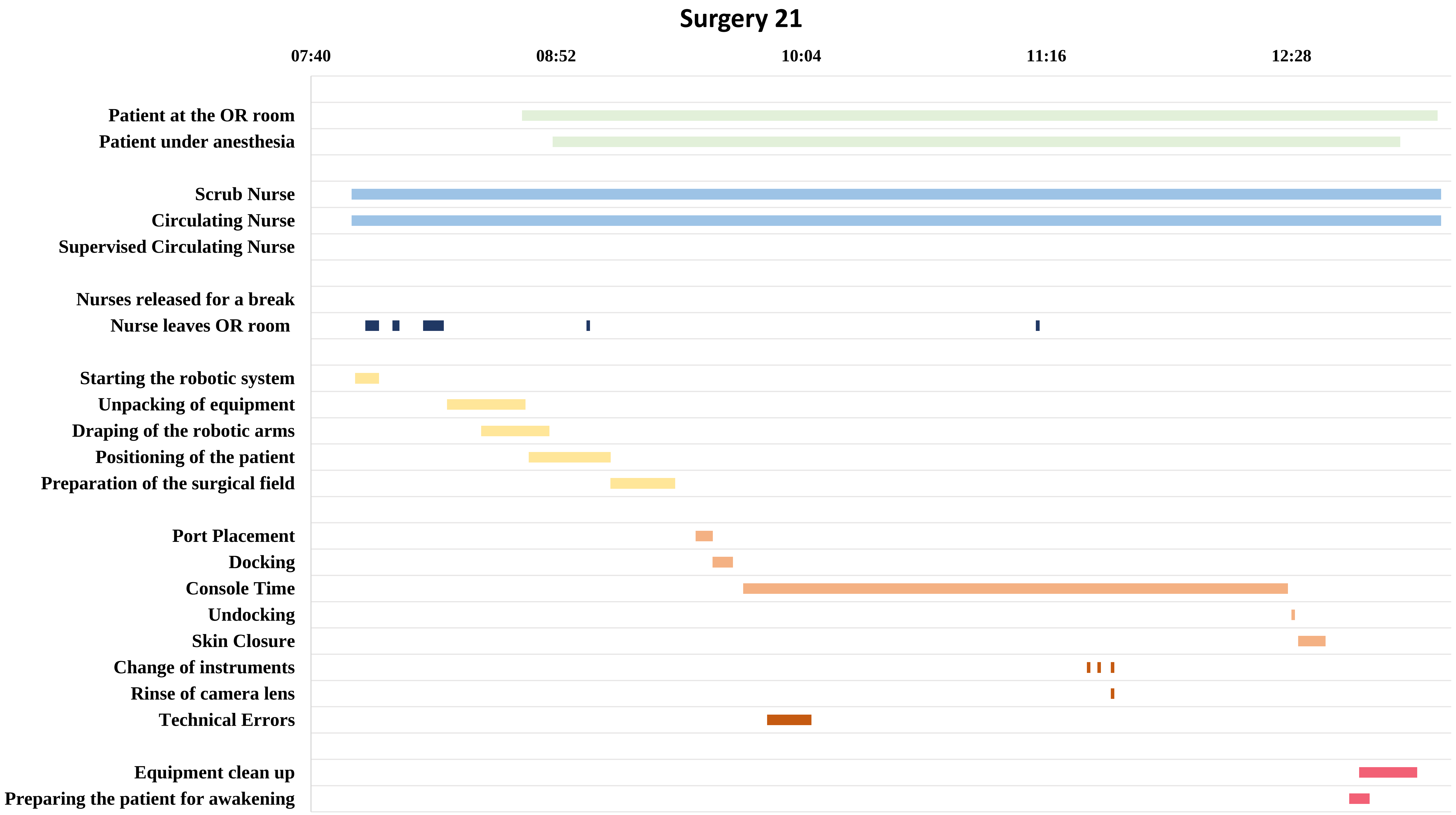
Surgery 21
07:40
08:52
10:04
11:16
12:28
Patient at the OR room
Patient under anesthesia
Scrub Nurse
Circulating Nurse
Supervised Circulating Nurse
Nurses released for a break
Nurse leaves OR room
Starting the robotic system
Unpacking of equipment
Draping of the robotic arms
Positioning of the patient
Preparation of the surgical field
Port Placement
Docking
Console Time
Undocking
Skin Closure
Change of instruments
Rinse of camera lens
Technical Errors
Equipment clean up
Preparing the patient for awakening

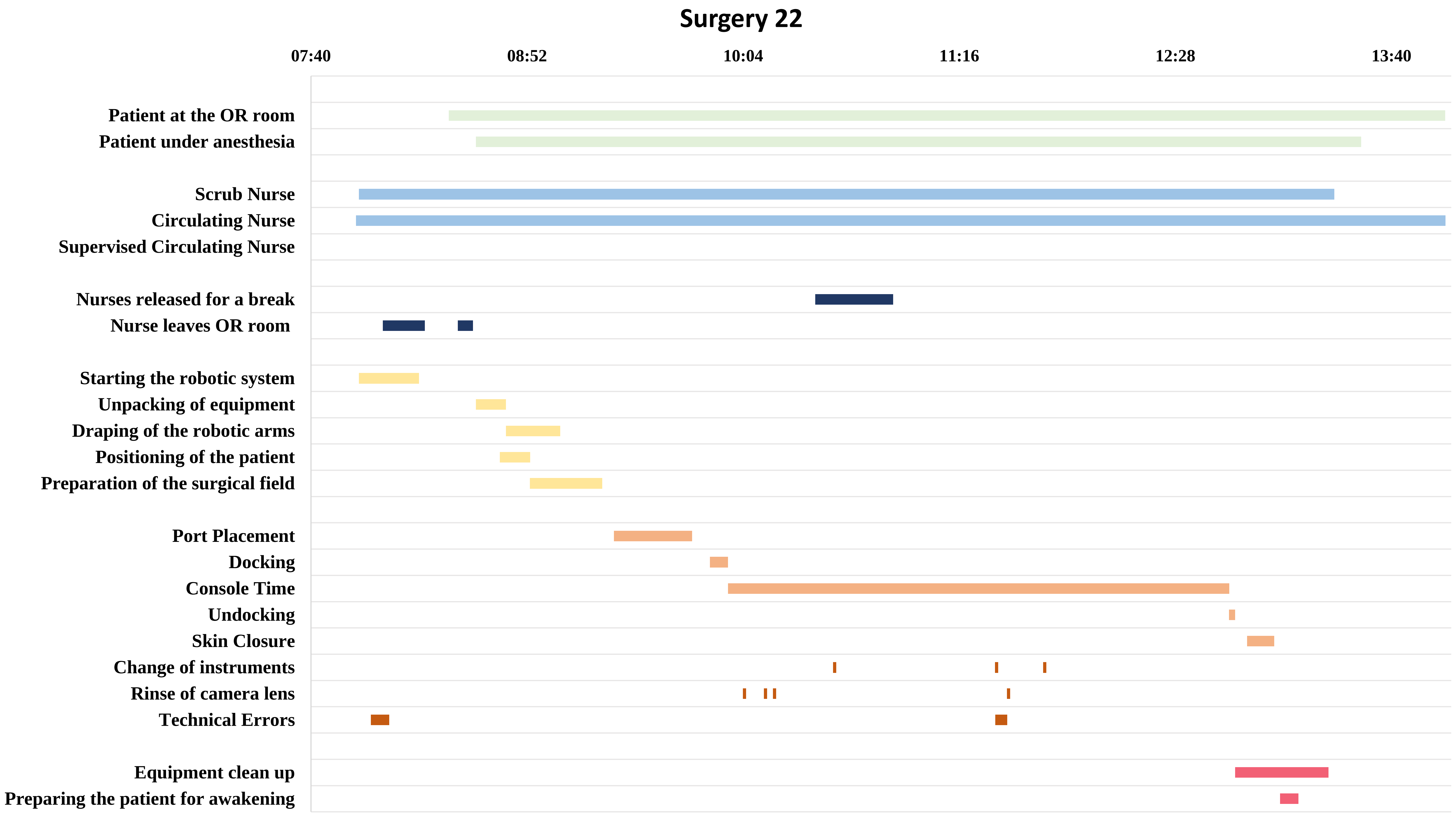

Surgery 22
07:40
08:52
10:04
11:16
12:28
13:40
Patient at the OR room
Patient under anesthesia
Scrub Nurse
Circulating Nurse
Supervised Circulating Nurse
Nurses released for a break
Nurse leaves OR room
Starting the robotic system
Unpacking of equipment
Draping of the robotic arms
Positioning of the patient
Preparation of the surgical field
Port Placement
Docking
Console Time
Undocking
Skin Closure
Change of instruments
Rinse of camera lens
Technical Errors
Equipment clean up
Preparing the patient for awakening

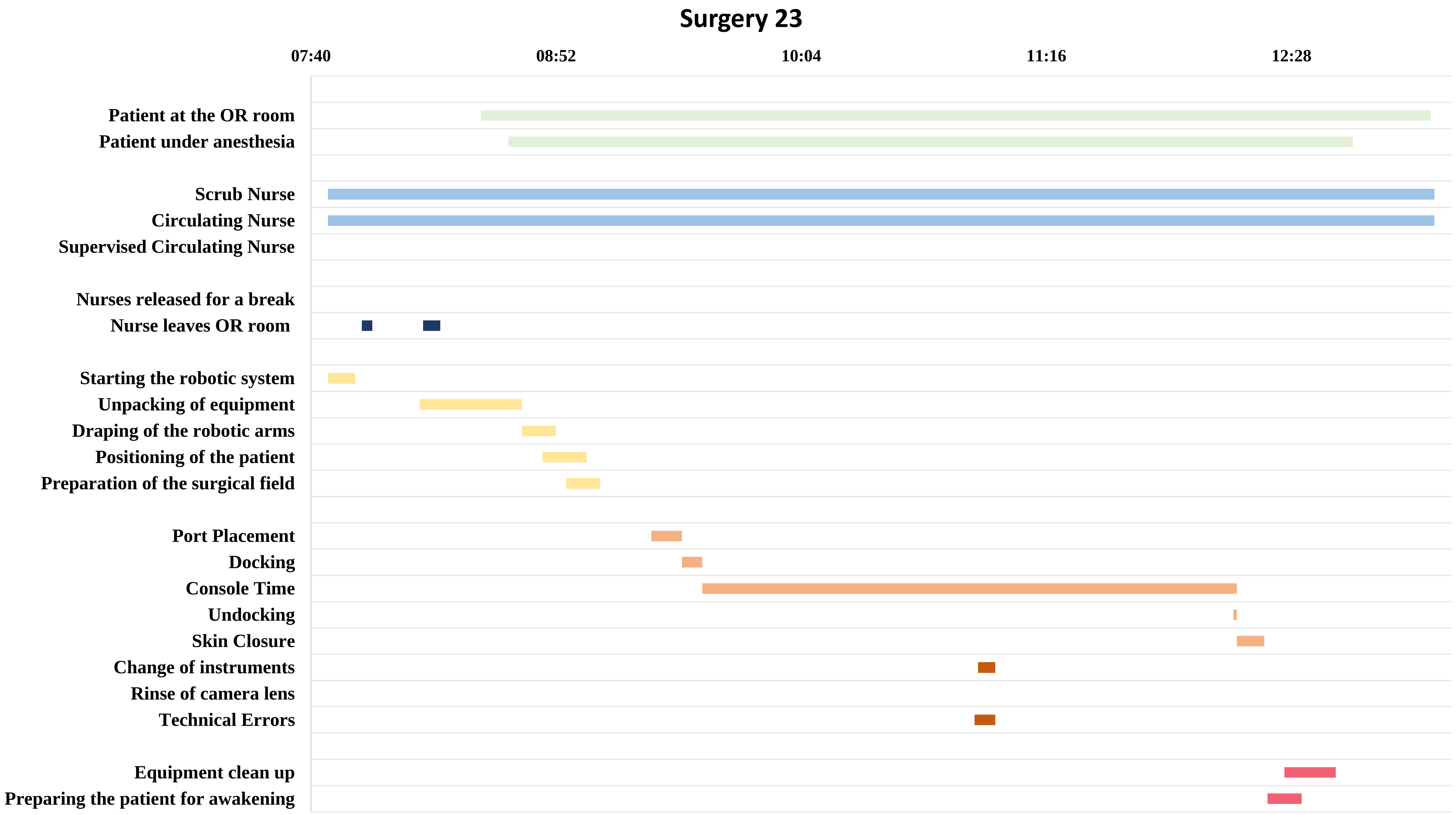
Surgery 23
07:40
08:52
10:04
11:16
12:28
Patient at the OR room
Patient under anesthesia
Scrub Nurse
Circulating Nurse
Supervised Circulating Nurse
Nurses released for a break
Nurse leaves OR room
Starting the robotic system
Unpacking of equipment
Draping of the robotic arms
Positioning of the patient
Preparation of the surgical field
Port Placement
Docking
Console Time
Undocking
Skin Closure
Change of instruments
Rinse of camera lens
Technical Errors
Equipment clean up
Preparing the patient for awakening

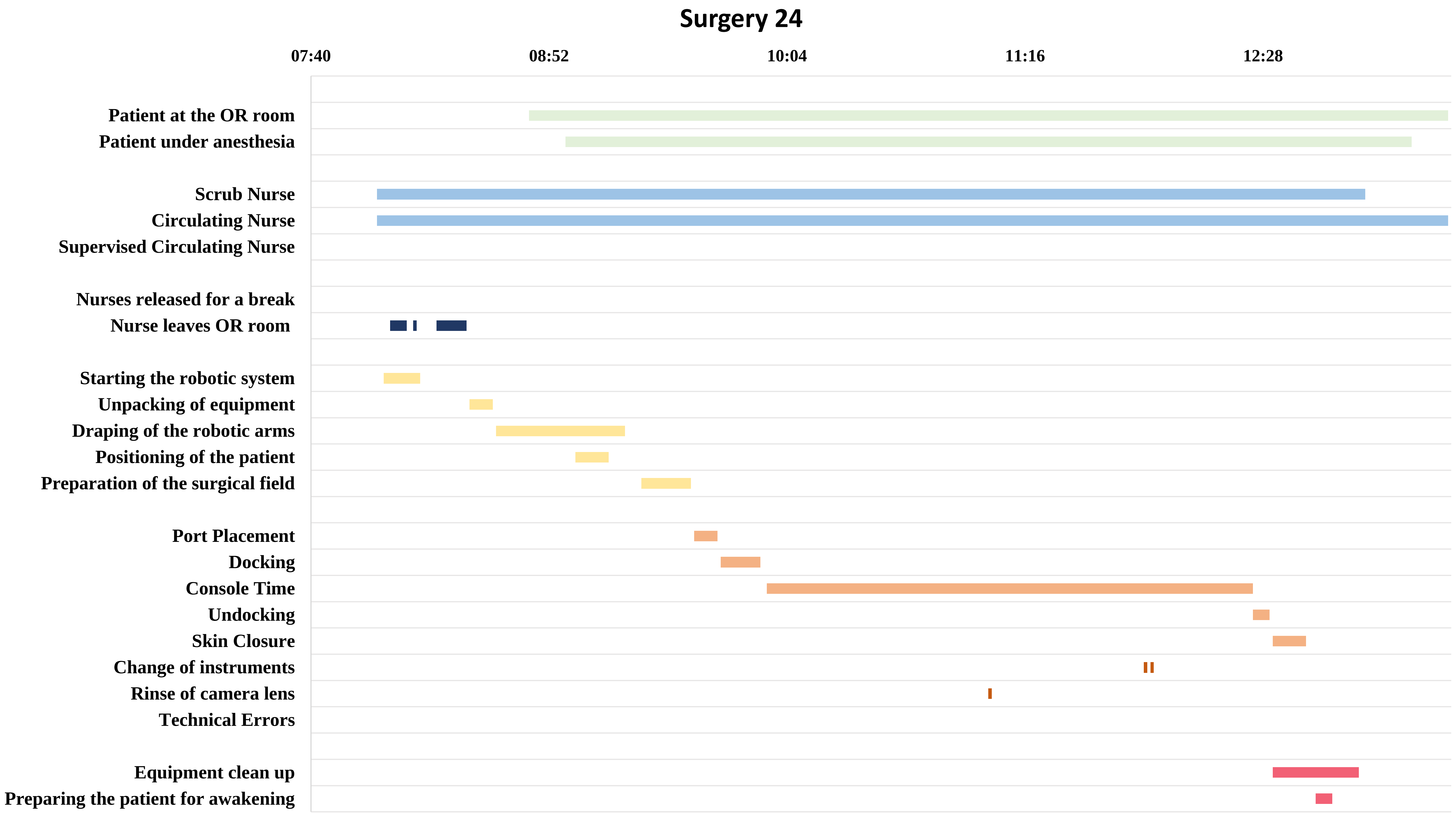
Surgery 24
07:40
08:52
10:04
11:16
12:28
Patient at the OR room
Patient under anesthesia
Scrub Nurse
Circulating Nurse
Supervised Circulating Nurse
Nurses released for a break
Nurse leaves OR room
Starting the robotic system
Unpacking of equipment
Draping of the robotic arms
Positioning of the patient
Preparation of the surgical field
Port Placement
Docking
Console Time
Undocking
Skin Closure
Change of instruments
Rinse of camera lens
Technical Errors
Equipment clean up
Preparing the patient for awakening

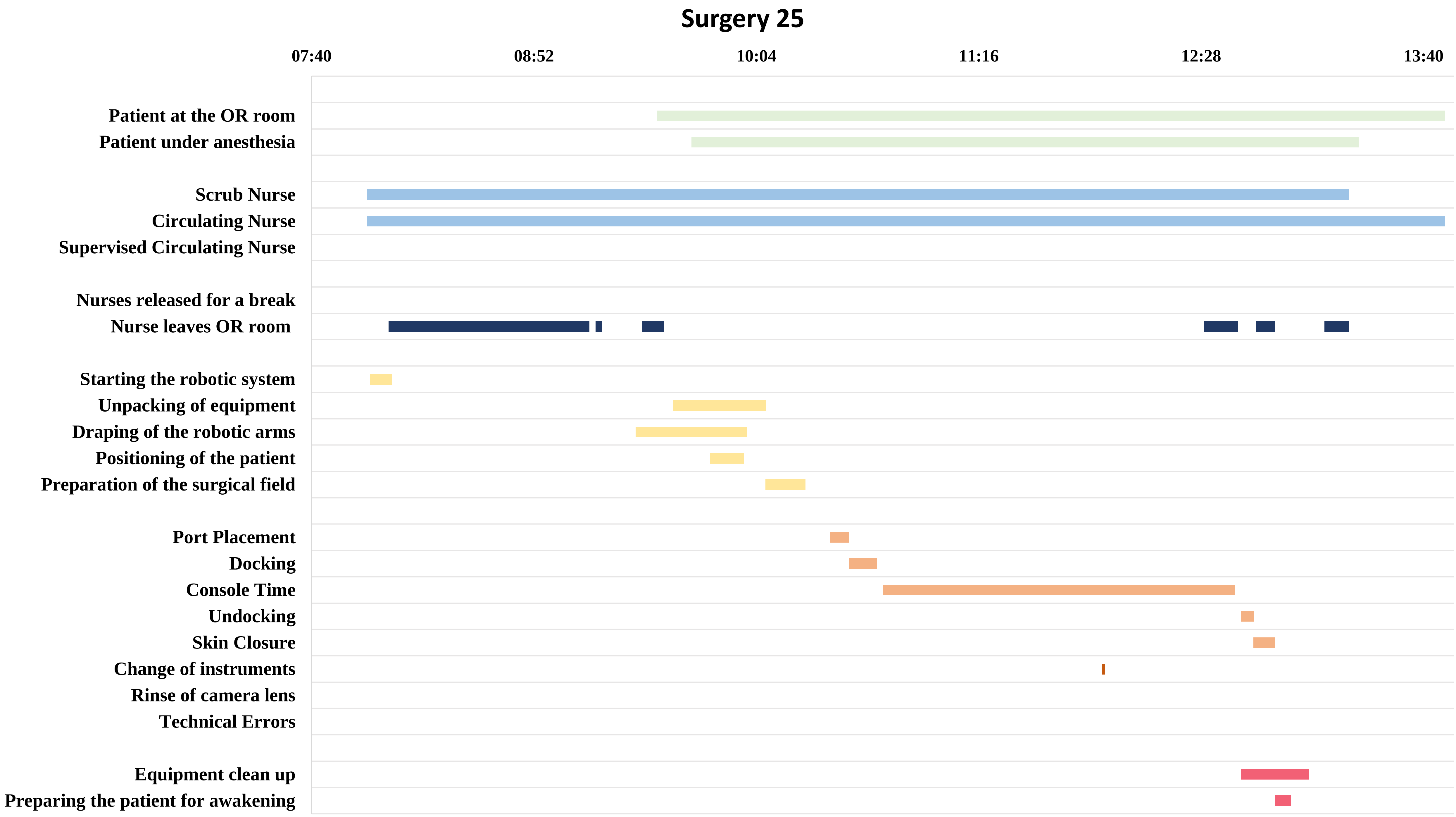
Surgery 25
07:40
08:52
10:04
11:16
12:28
13:40
Patient at the OR room
Patient under anesthesia
Scrub Nurse
Circulating Nurse
Supervised Circulating Nurse
Nurses released for a break
Nurse leaves OR room
Starting the robotic system
Unpacking of equipment
Draping of the robotic arms
Positioning of the patient
Preparation of the surgical field
Port Placement
Docking
Console Time
Undocking
Skin Closure
Change of instruments
Rinse of camera lens
Technical Errors
Equipment clean up
Preparing the patient for awakening

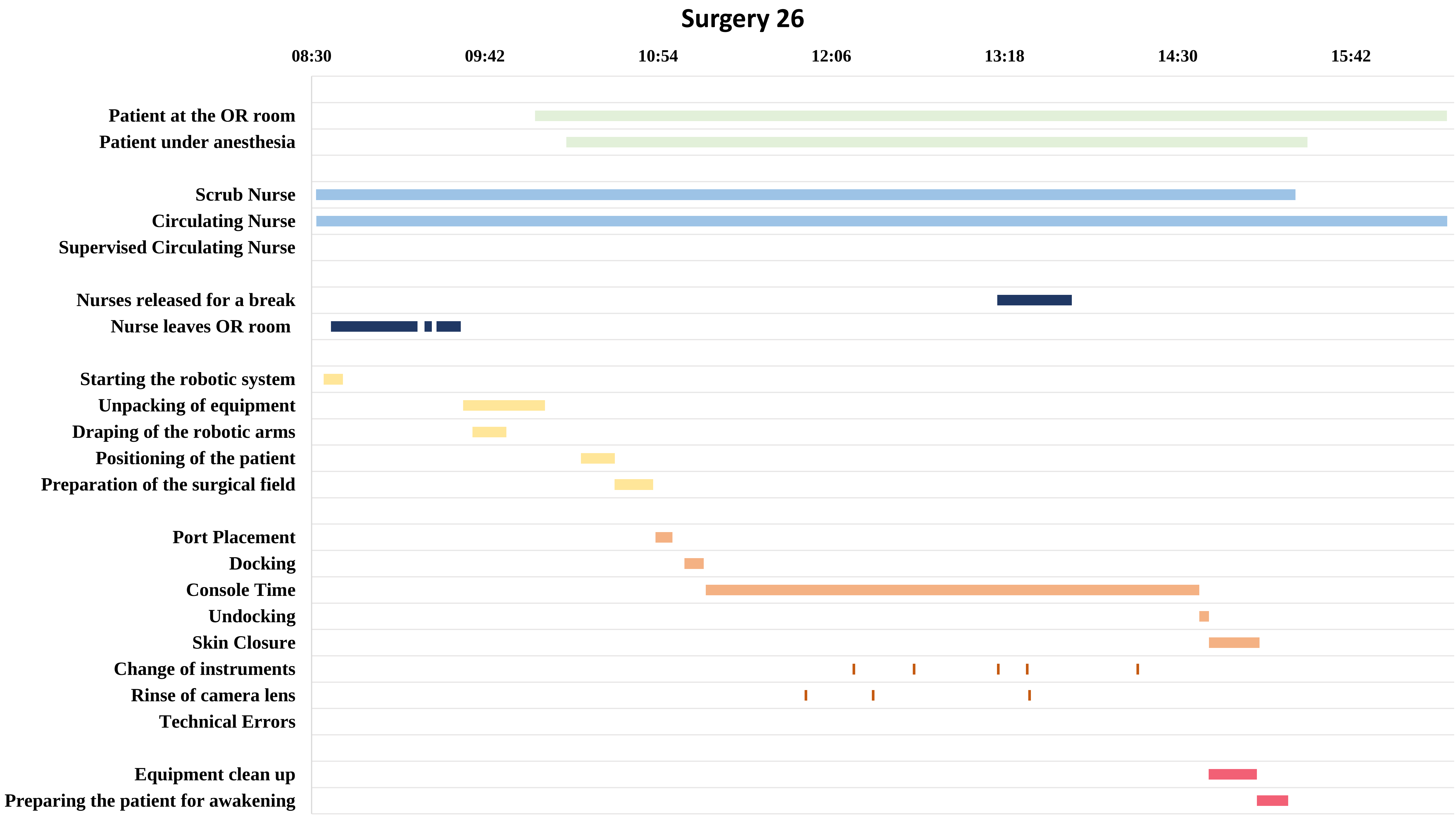
Surgery 26
08:30
09:42
10:54
12:06
13:18
14:30
15:42
Patient at the OR room
Patient under anesthesia
Scrub Nurse
Circulating Nurse
Supervised Circulating Nurse
Nurses released for a break
Nurse leaves OR room
Starting the robotic system
Unpacking of equipment
Draping of the robotic arms
Positioning of the patient
Preparation of the surgical field
Port Placement
Docking
Console Time
Undocking
Skin Closure
Change of instruments
Rinse of camera lens
Technical Errors
Equipment clean up
Preparing the patient for awakening

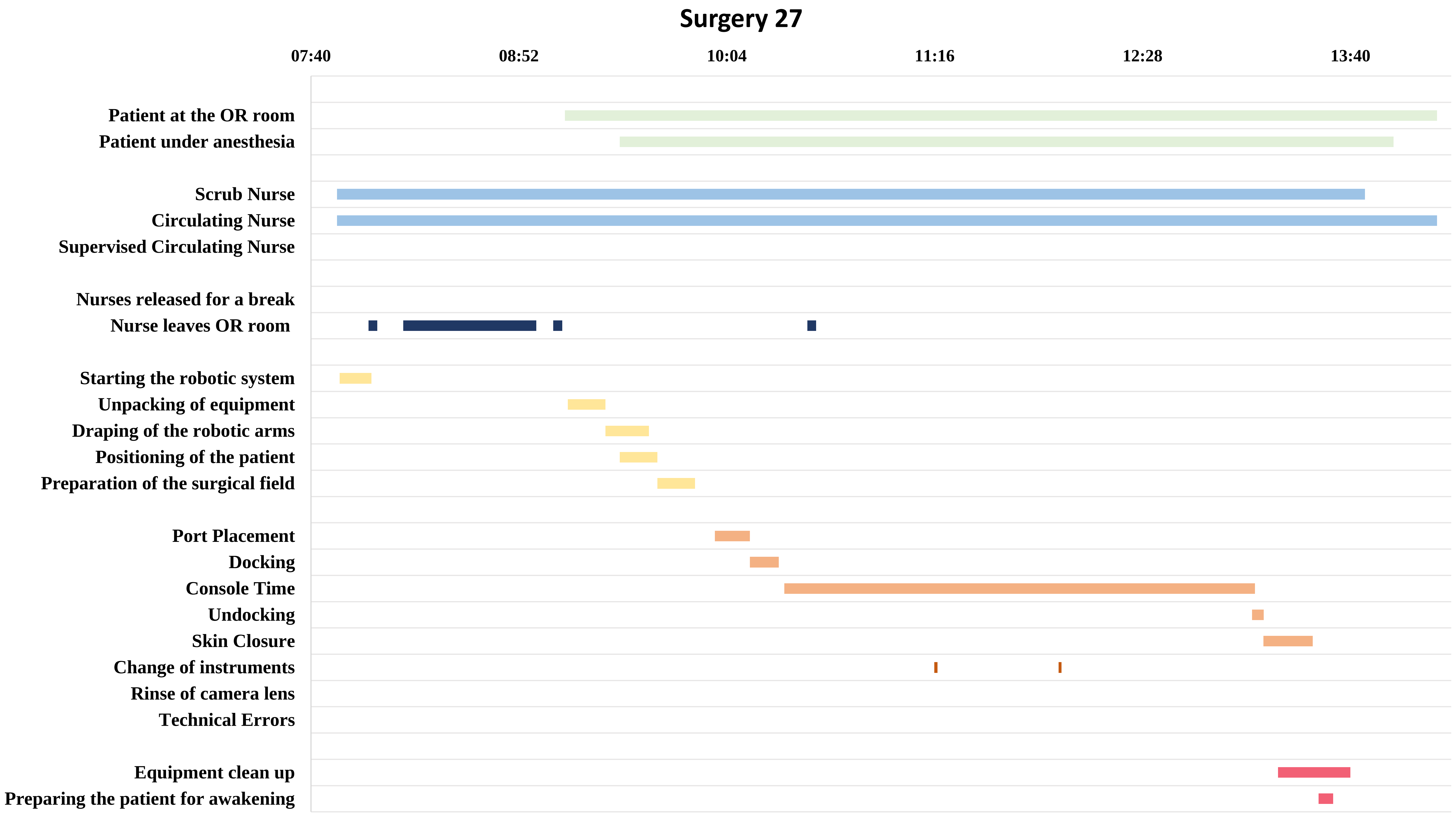
Surgery 27
07:40
08:52
10:04
11:16
12:28
13:40
Patient at the OR room
Patient under anesthesia
Scrub Nurse
Circulating Nurse
Supervised Circulating Nurse
Nurses released for a break
Nurse leaves OR room
Starting the robotic system
Unpacking of equipment
Draping of the robotic arms
Positioning of the patient
Preparation of the surgical field
Port Placement
Docking
Console Time
Undocking
Skin Closure
Change of instruments
Rinse of camera lens
Technical Errors
Equipment clean up
Preparing the patient for awakening

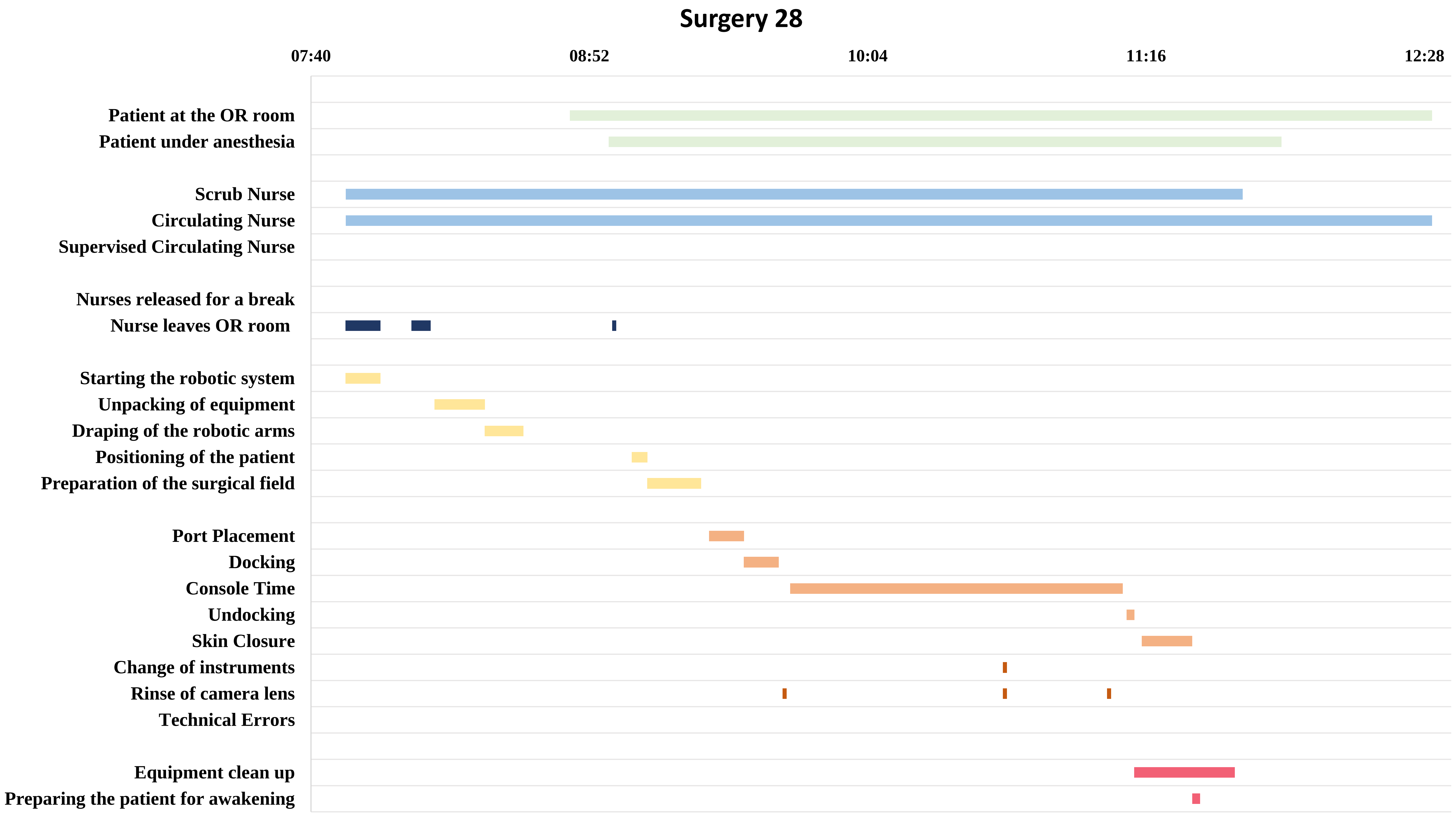
Surgery 28
07:40
08:52
10:04
11:16
12:28
Patient at the OR room
Patient under anesthesia
Scrub Nurse
Circulating Nurse
Supervised Circulating Nurse
Nurses released for a break
Nurse leaves OR room
Starting the robotic system
Unpacking of equipment
Draping of the robotic arms
Positioning of the patient
Preparation of the surgical field
Port Placement
Docking
Console Time
Undocking
Skin Closure
Change of instruments
Rinse of camera lens
Technical Errors
Equipment clean up
Preparing the patient for awakening

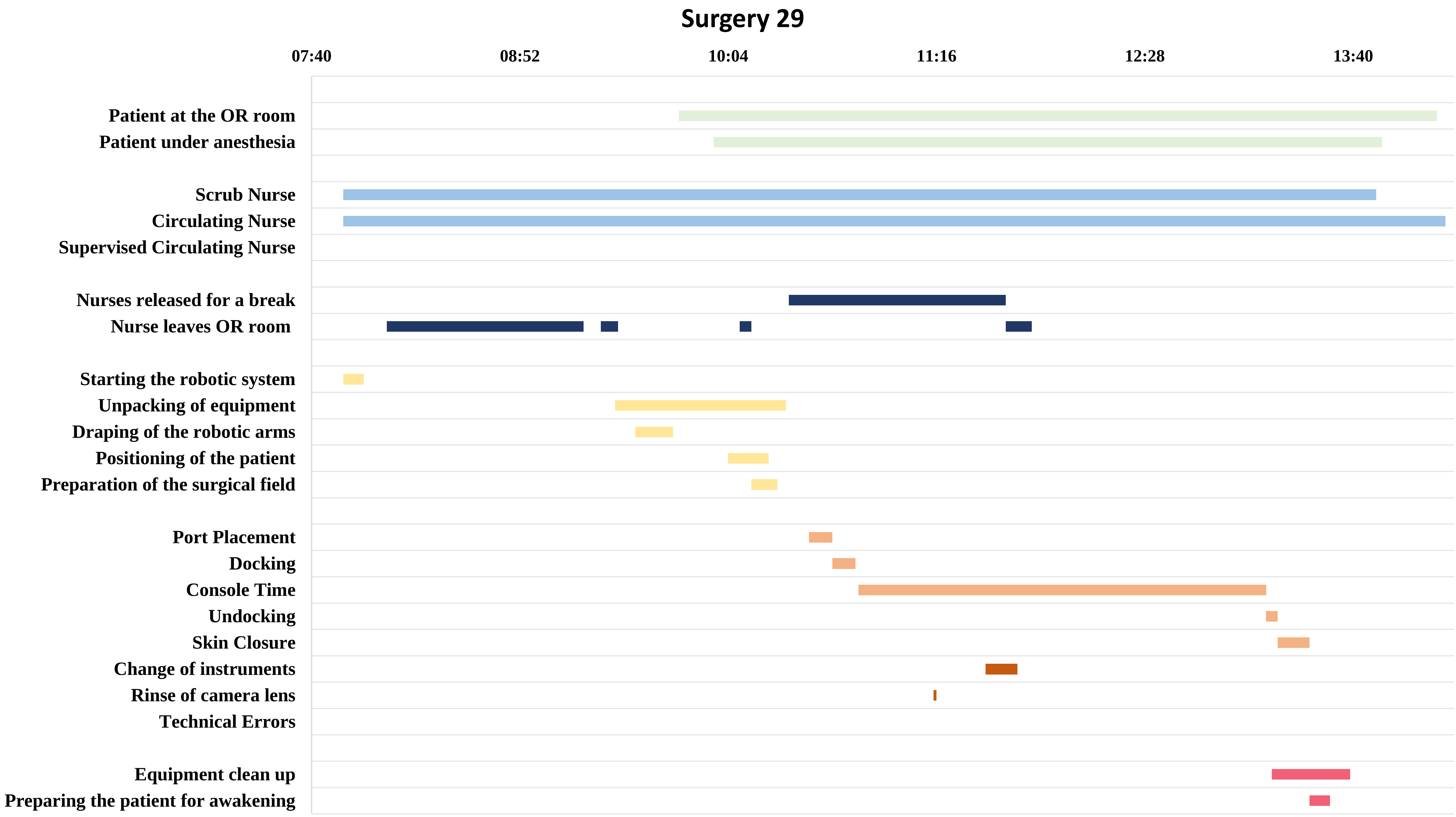
Surgery 29
07:40
08:52
10:04
11:16
12:28
13:40
Patient at the OR room
Patient under anesthesia
Scrub Nurse
Circulating Nurse
Supervised Circulating Nurse
Nurses released for a break
Nurse leaves OR room
Starting the robotic system
Unpacking of equipment
Draping of the robotic arms
Positioning of the patient
Preparation of the surgical field
Port Placement
Docking
Console Time
Undocking
Skin Closure
Change of instruments
Rinse of camera lens
Technical Errors
Equipment clean up
Preparing the patient for awakening

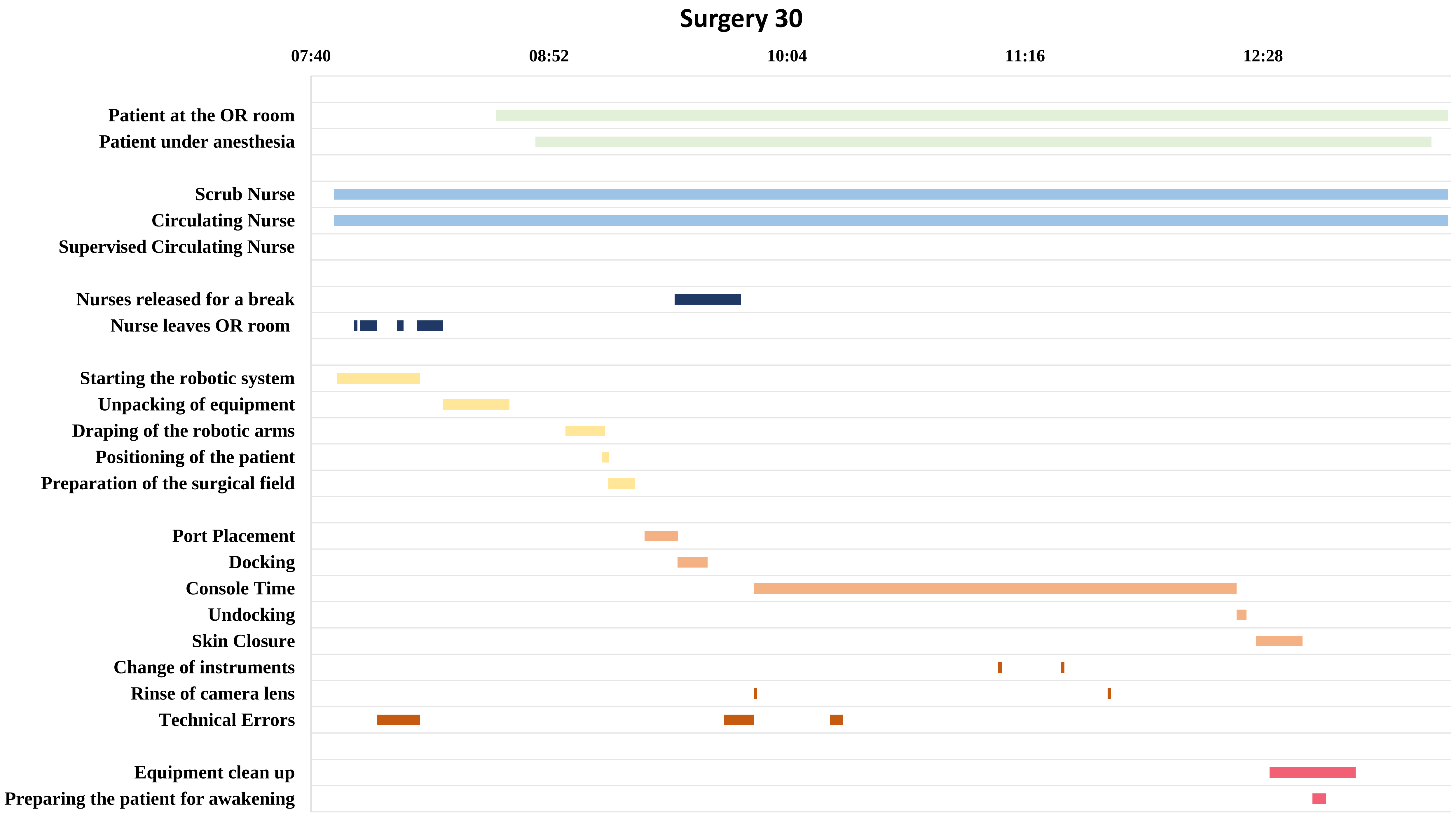

Surgery 30
07:40
08:52
10:04
11:16
12:28
Patient at the OR room
Patient under anesthesia
Scrub Nurse
Circulating Nurse
Supervised Circulating Nurse
Nurses released for a break
Nurse leaves OR room
Starting the robotic system
Unpacking of equipment
Draping of the robotic arms
Positioning of the patient
Preparation of the surgical field
Port Placement
Docking
Console Time
Undocking
Skin Closure
Change of instruments
Rinse of camera lens
Technical Errors
Equipment clean up
Preparing the patient for awakening
